# SOX10-regulated promoter use defines isoform-specific gene expression in Schwann cells

**DOI:** 10.1186/s12864-020-06963-7

**Published:** 2020-08-08

**Authors:** Elizabeth A. Fogarty, Jacob O. Kitzman, Anthony Antonellis

**Affiliations:** 1 Neuroscience Graduate Program, University of Michigan, Ann Arbor, MI USA; 2 Department of Human Genetics, University of Michigan, Ann Arbor, MI USA; 3 Department of Computational Medicine and Bioinformatics, University of Michigan, Ann Arbor, MI USA; 4 Department of Neurology, University of Michigan, 3710A Medical Sciences II, 1241 E. Catherine St. SPC, Ann Arbor, MI 5618 USA

**Keywords:** Schwann cell, Myelination, Promoter, Isoform, SOX10

## Abstract

**Background:**

Multicellular organisms adopt various strategies to tailor gene expression to cellular contexts including the employment of multiple promoters (and the associated transcription start sites (TSSs)) at a single locus that encodes distinct gene isoforms. Schwann cells—the myelinating cells of the peripheral nervous system (PNS)—exhibit a specialized gene expression profile directed by the transcription factor SOX10, which is essential for PNS myelination. SOX10 regulates promoter elements associated with unique TSSs and gene isoforms at several target loci, implicating SOX10-mediated, isoform-specific gene expression in Schwann cell function. Here, we report on genome-wide efforts to identify SOX10-regulated promoters and TSSs in Schwann cells to prioritize genes and isoforms for further study.

**Results:**

We performed global TSS analyses and mined previously reported ChIP-seq datasets to assess the activity of SOX10-bound promoters in three models: (***i***) an adult mammalian nerve; (***ii***) differentiating primary Schwann cells, and (***iii***) cultured Schwann cells with ablated SOX10 function. We explored specific characteristics of SOX10-dependent TSSs, which provides confidence in defining them as SOX10 targets. Finally, we performed functional studies to validate our findings at four previously unreported SOX10 target loci: *ARPC1A*, *CHN2*, *DDR1*, and *GAS7*. These findings suggest roles for the associated SOX10-regulated gene products in PNS myelination.

**Conclusions:**

In sum, we provide comprehensive computational and functional assessments of SOX10-regulated TSS use in Schwann cells. The data presented in this study will stimulate functional studies on the specific mRNA and protein isoforms that SOX10 regulates, which will improve our understanding of myelination in the peripheral nerve.

## Background

The complexity of the mammalian genome—in particular the expression of multiple, unique products from a single locus—is critical for the development and function of specialized cell types. This complexity is realized through a variety of mechanisms including alternative splicing, alternative start codon usage, alternative polyadenylation, and alternative promoter use [1]. Comprehensive transcription start site (TSS) mapping across many cell types and tissues has shown that each human gene harbors an average of four distinct TSSs [2]. Importantly, 80% of TSSs are utilized in a restricted (i.e., non-ubiquitous) manner, indicating that regulation of TSS use is an important contributor to cell-specific gene expression. In support of this notion, it has been reported that differential use of transcription start and termination sites accounts for the majority of isoform diversity in mammalian genomes [3–5].

As the myelinating cells of the peripheral nervous system (PNS), Schwann cells mediate saltatory conduction of action potentials along PNS axons. Schwann cells also provide critical trophic and metabolic support for PNS axons throughout development and in adult animals. Deficits in Schwann cell function, including those associated with inherited demyelinating peripheral neuropathies, can impair sensory and motor function to the point of rendering affected individuals wheelchair-bound [6]. Despite the importance of Schwann cells for PNS function, they have not been extensively studied compared to other human cell types and much remains to be learned about Schwann cell biology. Importantly, identifying and characterizing genes important for PNS myelination is necessary for a more complete understanding of Schwann cell function and related human diseases.

The SRY-box transcription factor 10 (SOX10) is a transcriptional activator that is critical for the development and maintenance of Schwann cells. SOX10 interacts with DNA in a sequence-specific manner through a high-mobility group (HMG) DNA-binding domain and can bind to DNA as a monomer or as a dimer [7]. Moreover, SOX10 facilitates gene expression through a number of mechanisms including interacting with the mediator complex [8] and transcriptional elongation factors [9], and through the recruitment of chromatin remodelers [10] and histone modifying enzymes [11]. SOX10 is expressed early in the Schwann cell lineage, beginning in migrating neural crest cells, with expression persisting in fully differentiated myelinating Schwann cells [12]. Loss of SOX10 expression in Schwann cells—even after the completion of myelination—leads to demyelination [13]. Further, SOX10 target genes characterized in Schwann cells to date include genes that are critical for myelination and that have been implicated in demyelinating disease (e.g., *EGR2*, *PMP22*, and *MPZ*) [14–16]. Therefore, the identification of novel SOX10 target genes in Schwann cells can be considered a viable strategy toward gaining new insights into PNS myelination.

SOX10 functions at distal enhancers to induce the expression of target genes [15, 17, 18]. However, a genome-wide assessment of SOX10 binding in rat sciatic nerve revealed that SOX10 binds more frequently at proximal promoter regions of target genes compared to other myelin-related transcription factors [19]. Consistent with the importance of SOX10 in activating gene promoters, multiple studies have described SOX10 binding directly at the promoters of myelin-related genes [16, 20–22]. Furthermore, certain loci harbor SOX10-regulated alternative promoters that direct the expression of unique transcript and protein isoforms [23–25]. These findings raise important questions about isoform specificity at these loci and about the roles of the SOX10-regulated gene products in Schwann cell function. To date, the relationship between SOX10 function and variable TSS usage has not been explored.

Given the importance of regulated TSS use for specialized cellular gene expression profiles [5], the critical role of SOX10 for Schwann cell function, and the previous identification of SOX10-regulated promoters that direct isoform-specific gene expression, we sought to define SOX10-mediated promoter use in Schwann cells genome-wide. We reasoned that such a study will advance the field by generating a rich dataset of candidate SOX10 target transcripts that may be prioritized for further study. Here, we report on the identification of SOX10-regulated promoters by assessing TSS use in the context of: (***i***) an adult mammalian nerve; (***ii***) differentiating primary Schwann cells; and (***iii***) upon loss of SOX10 in cultured Schwann cells. We also perform functional studies to validate bona fide SOX10-regulated promoters at four loci identified by our efforts and discuss the relevance of the findings to Schwann cell biology. In sum, our data represent a prioritized functional classification of candidate SOX10 target genes and isoforms that will contribute to a more complete understanding of Schwann cells, myelination, and myelin-related disease phenotypes.

## Results

### SOX10 binds to promoters in Schwann cells in vivo

**Fig. 1:**
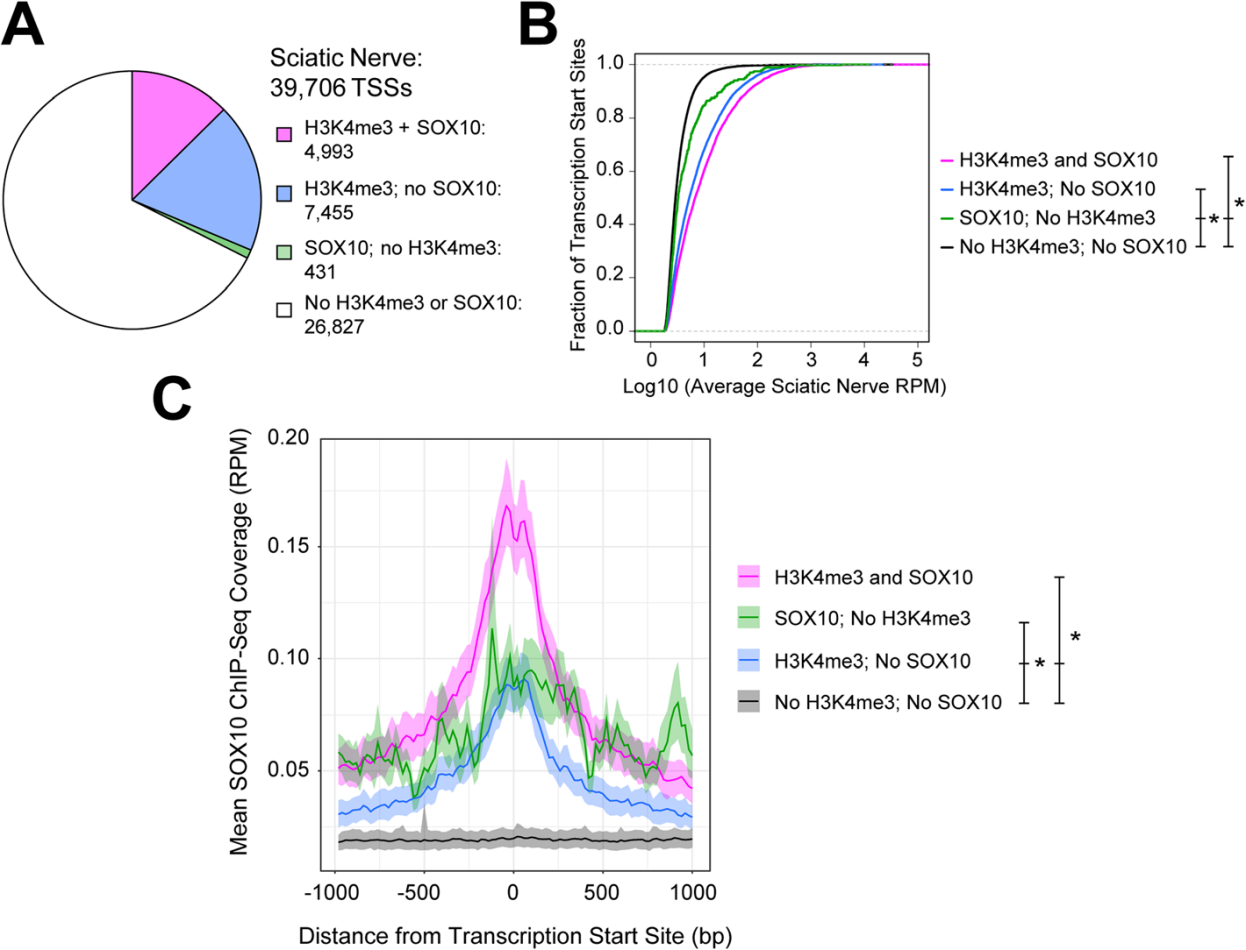
SOX10 binds to promoter elements in Schwann cells in vivo. **a** Tn5Prime-defined transcription start sites (TSSs) in sciatic nerve mapping within 1 kilobase of SOX10 and/or H3K4me3 ChIP-Seq peaks. **b** Expression levels of TSS classes (as in panel A). X-axis: log10 transformed average expression level (reads per million, RPM). Y-axis: the cumulative fraction of TSSs. Asterisk indicates *p* < 0.00001. **c** Aggregate SOX10 ChIP-Seq data in the 2-kilobase region surrounding TSS classes (as in panel A). X-axis: genomic distance from the TSS (base pairs, bp). Y-axis: average SOX10 ChIP-Seq signal (RPM, reads per million). Asterisk indicates *p* < 0.01

In Schwann cells, SOX10 regulates the promoters of critical myelin genes including loci that harbor more than one transcription start site (TSS) [16, 20, 21, 23]. This suggests that genome-wide characterization of SOX10 activity at promoter elements will reveal gene products important for PNS myelination. To define SOX10 binding at promoters in Schwann cells in vivo, we performed Tn5Prime library preparation [26] using RNA isolated from adult rat sciatic nerves (age 6–9 months) to define TSSs in this tissue. Tn5Prime identifies TSSs through the use of a 5′ anchored template-switching oligo during reverse transcription of total RNA. Size selection ensures that the sequenced library includes fragments derived from elongated transcripts greater than 200 bp in length, and Tn5Prime data have been validated to quantify transcript abundance in a similar manner as other sequencing-based methods [26]. After generating and mapping TSS data, we intersected them with published SOX10 and H3K4me3 ChIP-Seq datasets [27, 28] from rat sciatic nerve to assess the proximity of TSSs to SOX10 binding and active promoter elements, respectively. We found that 4993 of the 39,706 TSSs (12.6%) active in sciatic nerve reside within one kilobase of an H3K4me3 ChIP-Seq peak that overlaps a SOX10 ChIP-Seq peak; this dataset represents TSSs potentially regulated by SOX10-bound promoters (Fig. 1a, Supplementary Table 1). These 4993 TSSs map to 2993 unique loci, including previously characterized SOX10 target genes (e.g., *Mpz, Mbp,* and *Pmp22*). Ontology analysis of these loci shows an enrichment for gene products associated with ‘regulation of myelination’ (GO:0031641; FDR-corrected *p*-value = 0.0324). In addition, 7455 of the 39,706 TSSs (18.8%) expressed in sciatic nerve map to H3K4me3 ChIP-Seq peaks but not a SOX10 ChIP-Seq peak; 431 (1.1%) map to a SOX10 peak but not an H3K4me3 peak; and the remaining 26,827 TSSs (67.6%) do not map to an H3K4me3 peak or a SOX10 peak (Fig. 1a). The large number of TSSs defined by Tn5Prime that are not associated with an H3K4me3 ChIP-Seq peak is consistent with other TSS-mapping approaches identifying TSSs throughout gene bodies [29]. We find that TSSs not associated with an H3K4me3 mark: (***i***) are expressed at lower levels compared to those associated with promoter marks, with greater than 80% of these TSSs detected at fewer than 5 reads per million (Fig. 1b); and (***ii***) map to gene bodies at the same rate (greater than 90%) as TSSs that are associated with promoter marks (Table 1).

**Table 1:** Gene mapping rates of Tn5Prime-defined transcription start sites classified by associations with H3K4me3 and SOX10 ChIP-Seq peaks

Group	Number of TSSs	Number Within 1 kb of Gene on Same Strand	Percent Within 1 kb of Gene on Same Strand	Number of Unique Genes
H3K4me3 ChIP and SOX10 ChIP	4993	4698	94.09	2993
H3K4me3 ChIP; No SOX10 ChIP	7455	6918	92.80	4741
SOX10 ChIP; No H3K4me3 ChIP	431	392	90.95	225
No H3K4me3 ChIP and No SOX10 ChIP	26,827	24,867	92.69	7088
Total	39,706	36,875	92.87	10,000

To investigate the spatial relationship between SOX10 binding and TSSs, we performed a metagene analysis [30] to assess aggregate SOX10 ChIP-Seq signal [19] in a 2-kilobase window surrounding each class of TSS defined in Fig. 1a. For those TSSs residing near an H3K4me3 peak, the SOX10 ChIP-Seq signal is concentrated directly over the TSS (Fig. 1c). This finding is consistent with SOX10 proximally regulating transcript expression at these promoters. The 4993 TSSs associated with SOX10 and H3K4me3 ChIP-Seq peaks exhibit stronger SOX10 ChIP-Seq signal than those associated with only an H3K4me3 peak, as expected; this supports prioritization of these elements as highly confident candidate SOX10-regulated promoters. Therefore, we considered the 4993 TSSs associated with both H3K4me3 and SOX10 ChIP-Seq peaks as candidates of interest for further study.

To characterize the potential for the 4993 candidate SOX10-regulated TSSs to inform our understanding of isoform-specific gene expression in Schwann cells, we assessed the complexity of human RefSeq transcripts annotated for the 2993 loci associated with these TSSs. We found that 739 of the 2993 loci (25%) are annotated with at least two RefSeq transcripts originating from unique transcription start sites (Supplementary Table 2). Furthermore, 525 of the 2993 loci (17.5%) include multiple TSSs associated with transcripts that encode distinct protein-coding sequences (Supplementary Table 2). Taken as a whole, our data support the need to functionally characterize candidate SOX10 target promoters to delineate isoform-specific expression profiles relevant for myelinating Schwann cells.

### Defining SOX10 function at promoters in differentiating Schwann cells

Our Tn5Prime data collected from adult sciatic nerve are limited by the cellular heterogeneity of the sciatic nerve and the lack of developmental expression profiles for transcripts of interest. To address these limitations, we assayed TSS usage in a rat primary Schwann cell differentiation paradigm [31]. Briefly, cells were treated with CPT-cAMP in culture, which increases the expression of myelin proteins and reduces the expression of proteins associated with immature Schwann cells (Supplementary Figure 1). We generated and sequenced Tn5Prime libraries using RNA isolated from untreated and cAMP-treated cells, and focused our analysis on the expression profiles of the 4993 candidate TSSs associated with SOX10-bound promoters in sciatic nerve (see above). Importantly, SOX10 expression is not dependent upon cAMP stimulation in Schwann cells in vivo [32, 33], and we saw no difference in *Sox10* transcript expression between untreated and cAMP-treated cells (data not shown). However, we anticipated that changes in the expression and/or activity of factors known to act with SOX10 at a number of myelin genes, including EGR2 [15, 34] and CREB [22, 35], may lead to changes in SOX10 binding and/or activity and result in altered transcriptional output at SOX10-associated promoters.

**Fig. 2:**
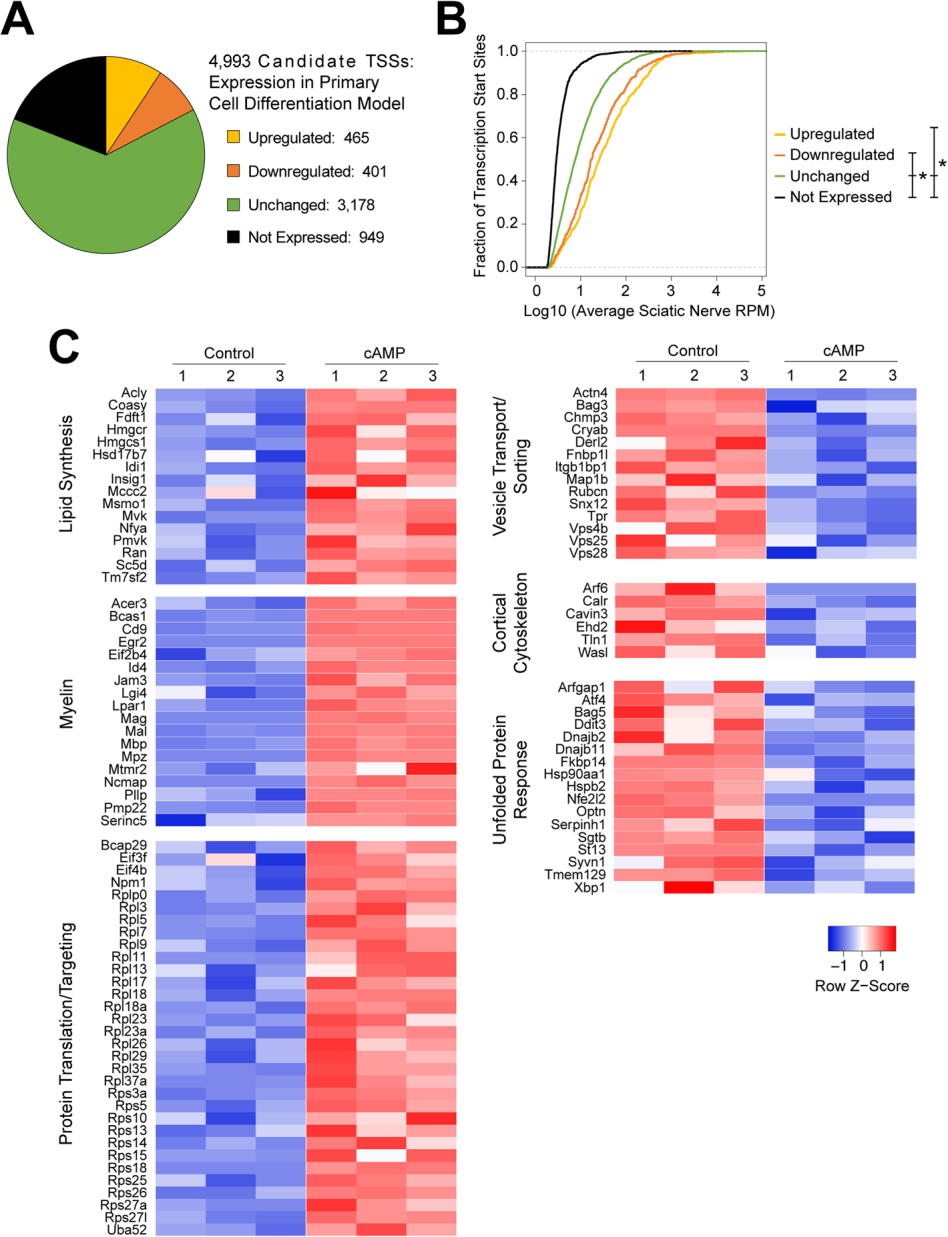
Assessment of SOX10-associated transcription start sites in differentiating primary Schwann cells. **a** Expression profiles of TSSs associated with SOX10-bound promoters in sciatic nerve, measured in control- and cAMP-treated primary Schwann cells. Upregulated by cAMP (yellow), downregulated by cAMP (orange), unchanged (green), or not expressed in either condition (black). **b** In vivo expression levels of TSS classes (as in panel **a**). X-axis: log10 transformed average expression level (reads per million, RPM). Y-axis: the cumulative fraction of TSSs. Asterisk indicates *p* < 0.03. **c** Per-sample expression levels for TSSs associated with enriched gene ontology terms among TSSs that are upregulated (left) and downregulated (right) upon cAMP treatment. Expression values are scaled per TSS (Z-score)

We first noted that 4044 of the 4993 candidate TSSs identified in sciatic nerve (81%) were expressed in control and/or cAMP-treated primary Schwann cells (Fig. 2a). Further, those TSSs that were not expressed by primary Schwann cells were among the most lowly-expressed TSSs in the sciatic nerve with greater than 80% detected at less than 5 reads per million in vivo (Fig. 2b). Of the 4044 TSSs expressed in this model, 465 (11.5%) exhibited increased expression with CPT-cAMP treatment while 401 (9.9%) showed reduced expression with treatment (Fig. 2a; Supplementary Table 1; for these and subsequent analyses, differential expression was defined by FDR-corrected *p*-value less than 0.05). These data provide cellular differentiation expression profiles for more than 4000 TSSs that are associated with SOX10-bound promoters and are especially relevant for the 866 TSSs with altered (up- or down-regulated) expression upon differentiation.

To assess the functional relevance of these findings, we performed gene ontology enrichment analyses on the upregulated and downregulated groups. The 465 upregulated TSSs map to 355 unique gene loci, and these genes are enriched for functional roles related to lipid synthesis, myelination, protein translation, and targeting of proteins to the endoplasmic reticulum and to the cell membrane (Table 2; Fig. 2c). These results not only support the validity of the differentiation model but also emphasize the presence of SOX10-bound promoters at broadly-expressed, “housekeeping” genes with functions that are particularly critical during active myelination such as lipid synthesis and protein targeting to the membrane. Conversely, the 401 downregulated TSSs map to 372 unique loci and are enriched for functions related to intracellular vesicle sorting and transport, cortical cytoskeleton regulation, and the unfolded protein response (Table 3; Fig. 2c). These findings suggest that the candidate SOX10 target transcripts associated with these functions may be most relevant for Schwann cell function at early developmental time points.

**Table 2:** Top 30 enriched gene ontology terms for transcription start sites that are upregulated upon primary Schwann cell differentiation, ranked by fold enrichment. Beneath each term is a list of the genes that contributed to the term’s enrichment

Gene Ontology Term	Fold Enrichment	FDR-Corrected P-Value
Isopentenyl diphosphate biosynthetic process (GO:0009240)	36.73	1.56E-02
*Idi1, Pmvk, Mvk*		
Isopentenyl diphosphate metabolic process (GO:0046490)	36.73	1.55E-02
*Idi1, Pmvk, Mvk*		
Ribosomal small subunit export from nucleus (GO:0000056)	26.23	2.99E-02
*Ran, Rps15, Npm1*		
Cholesterol biosynthetic process (GO:0006695)	17.49	1.55E-08
*Insig1, Hds17b7, Hmgcr, Msmo1, Hmgcs1, Idi1, Pmvk, Tm7sf2, Fdft1, Mvk, Acly, Sc5d*		
Secondary alcohol biosynthetic process (GO:1902653)	17.08	1.87E-08
*Insig1, Hsd17b7, Hmgcr, Msmo1, Hmgcs1, Idi1, Pmvk, Tm7sf2, Fdft1, Mvk, Acly, Sc5d*		
SRP-dependent cotranslational protein targeting to membrane (GO:0006614)	17.04	2.45E-19
*Rpl18a, Rps15, Rpl9, Rps10, Rpl37a, Rpl29, Rps3a, Rpl13, Rpl23a, Rpl17, Rpl11, Rpl23, Rps5, Rps14, Rps18, Uba52, Rpl35, Rps25, Rpl26, Rpl18, Rpl3, Rps27a, Rps13, Rpl7, Rps26, Rplp0, Rpl5*		
Coenzyme A metabolic process (GO:0015936)	16.32	1.52E-02
*Hmgcr, Mccc2, Coasy, Acly*		
Cotranslational protein targeting to membrane (GO:0006613)	16.2	5.64E-19
*Rpl18a, Rps15, Rpl9, Rps10, Rpl37a, Rpl29, Rps3a, Rpl13, Rpl23a, Rpl17, Rpl11, Rpl23, Rps5, Rps14, Rps18, Uba52, Rpl35, Rps25, Rpl26, Rpl18, Rpl3, Rps27a, Rps13, Rpl7, Rps26, Rplp0, Rpl5*		
Ribosomal small subunit assembly (GO:0000028)	15.3	3.80E-03
*Rps15, Rps10, Rps5, Rps14, Rps27l*		
Sterol biosynthetic process (GO:0016126)	15.3	4.86E-08
*Insig1, Hsd17b7, Hmgcr, Msmo1, Hmgcs1, Idi1, Pmvk, Tm7sf2, Fdft1, Mvk, Acly, Sc5d*		
Protein targeting to ER (GO:0045047)	15.02	2.04E-18
*Rpl18a, Rps15, Rpl9, Rps10, Rpl37a, Rpl29, Rps3a, Rpl13, Rpl23a, Rpl17, Rpl11, Rpl23, Rps5, Rps14, Rps18, Uba52, Rpl35, Rps25, Rpl26, Rpl18, Rpl3, Rps27a, Rps13, Rpl7, Rps26, Rplp0, Rpl5*		
Viral transcription (GO:0019083)	14.9	5.06E-19
*Rpl18a, Rps15, Rpl9, Rps10, Rpl37a, Rpl29, Rps3a, Rpl13, Rpl23a, Rpl17, Rpl11, Rpl23, Rps5, Rps14, Rps18, Uba52, Rpl35, Rps25, Rpl26, Rpl18, Rpl3, Rps27a, Rps13, Rpl7, Rps26, Rplp0, Seh1l, Rpl5*		
Establishment of protein localization to endoplasmic reticulum (GO:0072599)	14.5	3.04E-18
*Rpl18a, Rps15, Rpl9, Rps10, Rpl37a, Rpl29, Rps3a, Rpl13, Rpl23a, Rpl17, Rpl11, Rpl23, Rps5, Rps14, Rps18, Uba52, Rpl35, Rps25, Rpl26, Rpl18, Rpl3, Rps27a, Rps13, Rpl7, Rps26, Rplp0, Rpl5*		
Regulation of cholesterol biosynthetic process (GO:0045540)	14.24	2.22E-06
*Ran, Hmgcr, Hmgcs1, Idi1, Nfya, Pmvk, Tm7sf2, Fdft1, Mvk, Sc5d*		
Regulation of sterol biosynthetic process (GO:0106118)	14.24	2.19E-06
*Ran, Hmgcr, Hmgcs1, Idi1, Nfya, Pmvk, Tm7sf2, Fdft1, Mvk, Sc5d*		
Viral gene expression (GO:0019080)	14.24	2.34E-19
*Rpl18a, Rps15, Rpl9, Pcbp2, Rps10, Rpl37a, Rpl29, Rps3a, Rpl13, Rpl23a, Rpl17, Rpl11, Rpl23, Rps5, Rps14, Rps18, Uba52, Rpl35, Rps25, Rpl26, Rpl18, E1f3f, Rpl3, Rps27a, Rps13, Rpl7, Rps26, Rplp0, Seh1l, Rpl5*		
Nuclear-transcribed mRNA catabolic process, nonsense-mediated decay (GO:0000184)	13.77	7.89E-18
*Rpl18a, Rps15, Rpl9, Rps10, Rpl37a, Rpl29, Rps3a, Rpl13, Rpl23a, Rpl17, Rpl11, Rpl23, Rps5, Rps14, Rps18, Uba52, Rpl35, Rps25, Rpl26, Rpl18, Rpl3, Rps27a, Rps13, Rpl7, Rps26, Rplp0, Rpl5*		
Isoprenoid biosynthetic process (GO:0008299)	13.12	1.47E-03
*Hmgcr, Hmgcs1, Idi1, Pmvk, Mvk*		
Translational initiation (GO:0006413)	13	2.20E-19
*Eif2b4, Rpl18a, Rps15, Rpl9, Rps10, Rpl37a, Rpl29, Rps3a, Rpl13, Eif4b, Rpl23a, Rpl17, Rpl11, Rpl23, Rps5, Rps14, Rps18, Uba52, Rpl35, Rps25, Rpl26, Rpl18, Eif3f, Rpl3, Rps27a, Eif1b, Rps13, Rpl7, Rps26, Rplp0*		
Protein localization to endoplasmic reticulum (GO:0070972)	12.96	1.83E-18
*Insig1, Rpl18a, Rps15, Rpl9, Rps10, Rpl37a, Rpl29, Rps3a, Rpl13, Rpl23a, Rpl17, Rpl11, Rpl23, Rps5, Bcap29, Rps14, Rps18, Uba52, Rpl35, Rps25, Rpl26, Rpl18, Rpl3, Rps27a, Rps13, Rpl7, Rps26, Rplp0, Rpl5*		
Myelin assembly (GO:0032288)	12.89	2.98E-02
*Ncmap, Cd9, Mtmr2, Pmp22*		
Nucleotide-excision repair, DNA duplex unwinding (GO:0000717)	11.13	4.41E-02
*Uba52, Rps27a, Gtf2h1, Ddb1*		
Regulation of cholesterol metabolic process (GO:0090181)	10.93	1.50E-05
*Ran, Hmgcr, Hmgcs1, Idi1, Nfya, Pmvk, Tm7sf2, Fdft1, Mvk, Sc5d*		
Protein targeting to membrane (GO:0006612)	10.65	4.14E-16
*Rpl18a, Rps15, Rpl9, Rps10, Rpl37a, Rpl29, Rps3a, Rpl13, Rab8b, Rpl23a, Pmp22, Rpl17, Rpl11, Aldh3a2, Rpl23, Rps5, Rps14, Rps18, Uba52, Rpl35, Rps25, Rpl26, Rpl18, Rpl3, Rps27a, Rps13, Rpl7, Rps26, Rplp0, Rpl5*		
Glucose 6-phosphate metabolic process (GO:0051156)	10.65	4.94E-02
*G6pc3, Taldo1, Pgls, Gpi*		
Myelination (GO:0042552)	10.59	4.51E-10
*Eif2b4, Mbp, Lpar1, Bcas1, Serinc5, Lgi4, Ncmap, Pllp, Egr2, Mpz, Id4, Acer3, Cd9, Mag, Dhh, Jam3, Mtmr2, Pmp22, Mal*		
Ensheathment of neurons (GO:0007272)	10.39	5.84E-10
*Eif2b4, Mbp, Lpar1, Bcas1, Serinc5, Lgi4, Ncmap, Pllp, Egr2, Mpz, Id4, Acer3, Cd9, Mag, Dhh, Jam3, Mtmr2, Pmp22, Mal*		
Axon ensheathment (GO:0008366)	10.39	5.70E-10
*Eif2b4, Mbp, Lpar1, Bcas1, Serinc5, Lgi4, Ncmap, Pllp, Egr2, Mpz, Id4, Acer3, Cd9, Mag, Dhh, Jam3, Mtmr2, Pmp22, Mal*		
Ribosome assembly (GO:0042255)	10.05	8.16E-06
*Rps15, Rps10, Rpl23a, Rpl11, Rps5, Rps14, Rps27l, Npm1, Rpl3, Rplp0, Rpl5*		
Cytoplasmic translation (GO:0002181)	9.62	1.17E-05
*Rpl18a, Rpl9, Rpl29, Eif4b, Rpl17, Rpl11, Rpl26, Rpl18, Eif3f, Rps26, Rplp0*

**Table 3:** Top 30 enriched gene ontology terms for transcription start sites that are downregulated upon primary Schwann cell differentiation, ranked by fold enrichment. Beneath each term is a list of the genes that contributed to the term’s enrichment

Gene Ontology Term	Fold Enrichment	FDR-Corrected P-Value
Vesicle transport along actin filament (GO:0030050)	33.96	2.44E-02
Wasl, Fnbp1l, Actn4		
Regulation of protein folding (GO:1903332)	22.64	8.45E-03
*Bag5, Dnajb2, Sgtb, St13*		
Activation of MAPKKK activity (GO:0000185)	20.58	1.08E-02
*Traf7, Gadd45b, Gadd45a, Gadd45g*		
Response to laminar fluid shear stress (GO:0034616)	18.86	2.87E-03
*Klf2, Xbp1, Nfe2l2, Ets1, Adam9*		
Establishment of protein localization to mitochondrial membrane (GO:0090151)	13.32	3.33E-02
*Hsp90aa1, Tomm22, Timm10*		
Membrane protein intracellular domain proteolysis (GO:0031293)	12.58	3.83E-02
*Aph1a, Traf6, Aph1b, Ngfr*		
Membrane protein proteolysis (GO:0033619)	10.71	1.80E-03
*Aph1a, Traf6, Aph1b, Adam19, Ngfr, Ctsh, Adam9*		
Response to fluid shear stress (GO:0034405)	9.99	7.03E-03
*Klf2, Xbp1, Cited2, Nfe2l2, Ets1, Adam9*		
Multivesicular body sorting pathway (GO:0071985)	8.84	3.32E-02
*Rubcn, Vps25, Vps28, Vps4b, Chmp3*		
Positive regulation of transcription from RNA polymerase II promoter in response to stress (GO:0036003)	8.84	3.30E-02
*Klf2, Xbp1, Nfe2l2, Atf4, Ddit3*		
Interleukin-12-mediated signaling pathway (GO:0035722)	8.61	4.59E-03
*P4hb, Capza1, Hnrnpdl, Cfl1, Rala, Rplp0, Pak2*		
Cortical actin cytoskeleton organization (GO:0030866)	8.49	1.36E-02
*Calr, Cavin3, Arf6, Tln1, Ehd2*		
Cellular response to interleukin-12 (GO:0071349)	8.25	5.46E-03
*P4hb, Capza1, Hnrnpdl, Cfl1, Rala, Rplp0, Pak2*		
Response to interleukin-12 (GO:0070671)	8.08	6.06E-03
*P4hb, Capza1, Hnrnpdl, Cfl1, Rala, Rplp0, Pak2*		
ER-nucleus signaling pathway (GO:0006984)	7.86	4.74E-02
*Calr, Xbp1, Nfe2l2, Atf4, Ddit3*		
Cortical cytoskeleton organization (GO:0030865)	7.55	2.25E-02
*Calr, Cavin3, Arf6, Rhoc, Tln1, Ehd2*		
Response to epidermal growth factor (GO:0070849)	6.93	3.03E-02
*Tpr, Col1a1, Gstp1, Zfp36, Snai2, Mars*		
Response to unfolded protein (GO:0006986)	6.41	2.32E-06
*Hspb2, Hsp90aa1, Dnajb2, Calr, Serpinh1, Derl2, Dnajb11, Xbp1, Arfgap1, Fkbp14, Nfe2l2, Atf4, Syvn1, Tmem129, Ddit3, Tln1, Bag3, Optn*		
Endoplasmic reticulum unfolded protein response (GO:0030968)	6.23	8.34E-04
*Calr, Derl2, Dnajb11, Xbp1, Arfgap1, Fkbp14, Nfe2l2, Atf4, Syvn1, Ddit3, Tln1*		
Negative regulation of intracellular transport (GO:0032387)	6.19	2.13E-02
*Cryab, Derl2, Tpr, Itgb1bp1, Map 1b, Bag3, Snx12*		
IRE1-mediated unfolded protein response (GO:0036498)	6.17	4.57E-02
*Dnajb11, Xbp1, Arfgap1, Fkbp14, Syvn1, Tln1*		
Cellular response to unfolded protein (GO:0034620)	6.03	2.09E-04
*Calr, Derl2, Dnajb11, Xbp1, Arfgap1, Fkbp14, Nfe2l2, Atf4, Syvn1, Ddit3, Tln1, Bag3, Optn*		
Intracellular steroid hormone receptor signaling pathway (GO:0030518)	5.74	2.91E-02
*Tada3, Calr, Ddx5, Ywhah, Ube3a, Nedd4, Plpp1*		
Response to topologically incorrect protein (GO:0035966)	5.6	9.46E-06
*Hspb2, Hsp90aa1, Dnajb2, Calr, Serpinh1, Derl2, Dnajb11, Xbp1, Arfgap1, Fkbp14, Nfe2l2, Atf4, Syvn1, Tmem129, Ddit3, Tln1, Bag3, Optn*		
Positive regulation of axonogenesis (GO:0050772)	5.33	2.01E-02
*Chd2, Map 2k1, Arhgdia, Tnfrsf12a, Eif4g2, Ngfr, Map 1b, Stk25*		
Regulation of transcription from RNA polymerase II promoter in response to stress (GO:0043618)	5.23	2.69E-03
*Klf2, Jun, Psmd8, Xbp1, Cited2, Nfe2l2, Atf4, Ddit3, Bag3, Nedd4, Higd1*a		
Cellular response to topologically incorrect protein (GO:0035967)	5.14	8.06E-04
*Calr, Derl2, Dnajb11, Xbp1, Arfgap1, Fkbp14, Nfe2l2, Atf4, Syvn1, Ddit3, Tln1, Bag3, Optn*		
Cell redox homeostasis (GO:0045454)	5.14	4.57E-02
*Nnt, P4hb, Pdia3, Prdx1, Nfe2l2, Nqo1, Ddit3, Tmx1*		
Endosome organization (GO:0007032)	5.14	4.55E-02
*Rab5b, Tmem127, Vps25, Vps28, Vps4b, Chmp3, Plekhj1*		
Rho protein signal transduction (GO:0007266)	5.08	4.76E-02
*Arhgdia, Cdh13, Cfl1, Ngfr, Adgrg1, Cdc42ep3*		

### A subset of SOX10-associated TSSs are dependent on SOX10

**Fig. 3:**
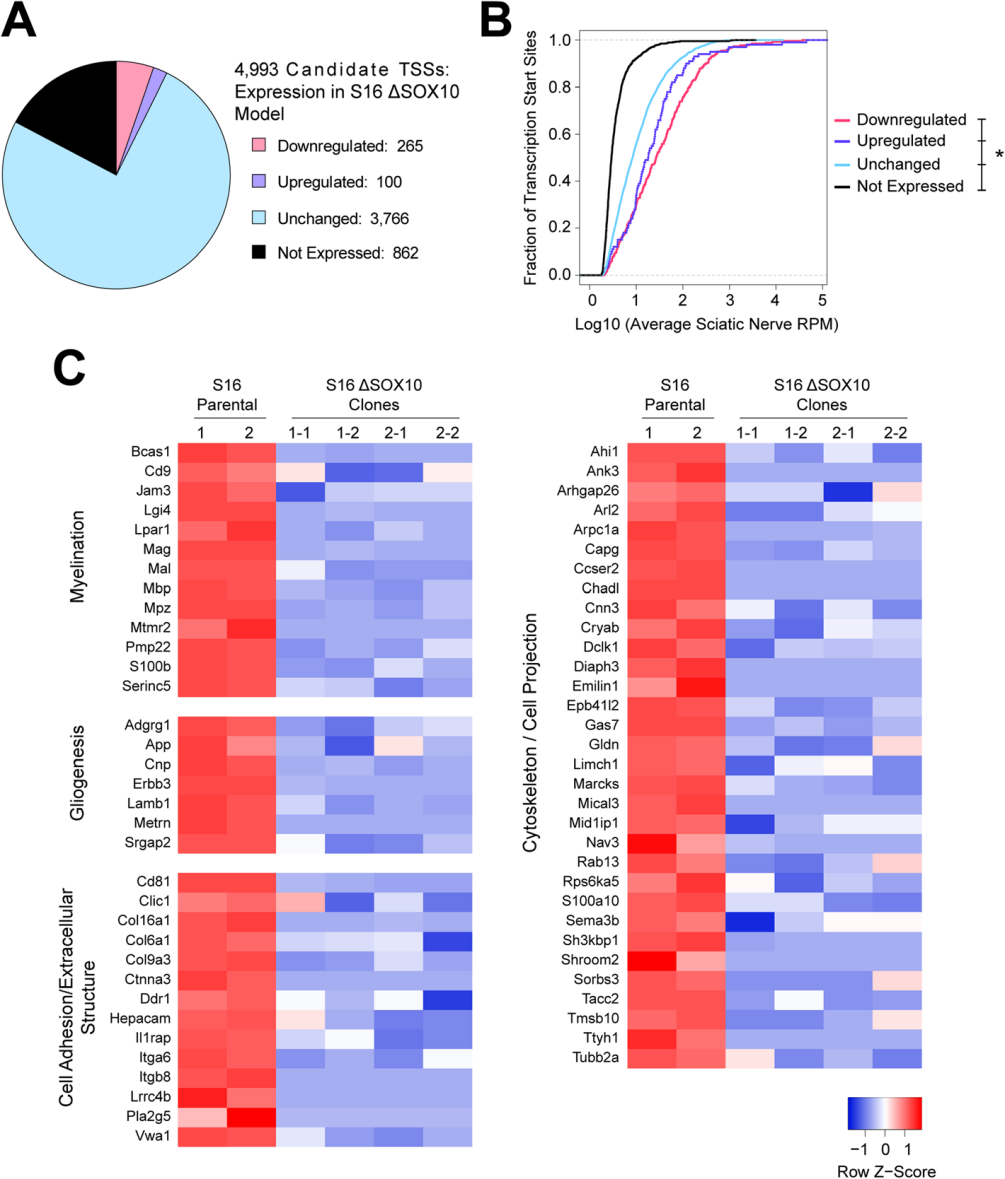
Assessment of SOX10-associated transcription start sites in ΔSOX10 S16 cells. **a** Expression profiles of TSSs associated with SOX10-bound promoters in sciatic nerve, measured in ΔSOX10 S16 cells compared to unmodified S16s. Downregulated by loss of SOX10 (pink), upregulated by loss of SOX10 (purple), unchanged (blue), or not expressed in either condition (black). **b** In vivo expression levels of TSS classes (as in panel A). X-axis: log10 transformed average expression level (reads per million, RPM). Y-axis: the cumulative fraction of TSSs. Asterisk indicates *p* < 0.05. **c** Per-sample expression levels for TSSs associated with enriched gene ontology terms among TSSs that are downregulated upon loss of SOX10. Expression values are scaled per TSS (Z-score)

To prioritize the 4993 SOX10-associated TSSs (see above) for those that represent bona fide SOX10-dependent target transcripts, we measured the expression of each TSS upon ablation of SOX10. We employed a rat myelinating Schwann cell line (S16 cells) [36] that expresses SOX10 and other markers of myelinating Schwann cells [37]. We generated S16 cells that lack SOX10 (ΔSOX10 S16 cells) via CRISPR/Cas9-mediated genome editing [38] (see methods and Supplementary Figure 2A). We recovered four clonal cell lines derived from two independent guide RNAs that each exhibit little or no *Sox10* transcript or protein (Supplementary Figure 2B and 2C). To assay the effect of SOX10 ablation on the expression of candidate SOX10-dependent TSSs, Tn5Prime libraries were generated using RNA collected from four individually isolated ΔSOX10 S16 clones and compared to libraries prepared from unmodified S16 cells. We focused our analysis on the 4993 candidate TSSs associated with SOX10-bound promoters in vivo. These data revealed that 265 of the 4993 candidate TSSs (5.3%) were downregulated in ΔSOX10 S16 cells compared to controls, consistent with dependence upon SOX10 for activation (Fig. 3a; Supplementary Table 1). Of the remaining TSSs, 100 (2.0%) were upregulated with loss of SOX10 (Supplementary Table 1), 3766 (75.4%) were unchanged, and 862 TSSs (17.3%) were not expressed in the S16 model (Fig. 3a). Similar to the primary Schwann cell model (Fig. 2b), the candidate TSSs that were not expressed in the S16 cell model included those that were most lowly expressed in vivo*,* again with greater than 80% detected at less than 5 reads per million in nerve (Fig. 3b). Given that SOX10 is understood to act primarily as a transcriptional activator [8, 19, 27, 39], we considered the TSSs that were downregulated upon loss of SOX10 as more likely resulting from direct transcriptional effects; therefore, we prioritized further study of these TSSs. In sum, the data from this model support the identification of 265 TSSs that are dependent on SOX10 in Schwann cells and that are regulated in a promoter-proximal manner.

To assess the functional significance of the SOX10-dependent TSSs we performed gene ontology enrichment analyses for this group. The 265 downregulated TSSs, which map to 169 unique genes, reside at loci associated with myelination and glial differentiation, as expected for SOX10 target genes (Table 4; Fig. 3c). Importantly, the enrichment for known SOX10-regulated pathways supports the validity of the model, as does comparison to previously published SOX10 knockdown experiments in S16 cells (Supplementary Table 3); in contrast, the above terms are not enriched among genes associated with TSSs that were upregulated or unchanged in ΔSOX10 S16 cells (data not shown). Interestingly, the 169 genes associated with downregulated TSSs are additionally enriched for functions related to cell adhesion, extracellular structure, cytoskeleton organization, and cellular projection (Table 4; Fig. 3c). Knockout of SOX10 has been previously associated with excessive deposition of extracellular matrix components [40] and increased cell adhesion [41], but the transcriptional mechanisms and target genes mediating these effects are unknown. Therefore, further study of the SOX10-regulated TSSs identified at genes in these functional groups will provide further insight into these processes in Schwann cells.

**Table 4:** Top 15 enriched gene ontology terms for transcription start sites that are downregulated upon loss of SOX10 in S16 cells, ranked by fold enrichment. Beneath each term is a list of the genes that contributed to the term’s enrichment

Gene Ontology Term	Fold Enrichment	FDR-Corrected P-Value
Regulation of myelination (GO:0031641)	17.54	1.41E-02
*Sox10, Lgi4, Mag, Ctsc, S100b, Mtmr2*		
Myelination (GO:0042552)	15.35	1.86E-07
*Mbp, Lpar1, Sox10, Bcas1, Serinc5, Lgi4, Mpz, Cd9, Mag, Dhh, Jam3, Mtmr2, Pmp22, Mal*		
Ensheathment of neurons (GO:0007272)	15.06	1.16E-07
*Mbp, Lpar1, Sox10, Bcas1, Serinc5, Lgi4, Mpz, Cd9, Mag, Dhh, Jam3, Mtmr2, Pmp22, Mal*		
Axon ensheathment (GO:0008366)	15.06	7.73E-08
*Mbp, Lpar1, Sox10, Bcas1, Serinc5, Lgi4, Mpz, Cd9, Mag, Dhh, Jam3, Mtmr2, Pmp22, Mal*		
Substantia nigra development (GO:0021762)	13.35	3.88E-02
*Mbp, Ndrg2, Mag, Cnp, Ldha*		
Gliogenesis (GO:0042063)	7.53	1.51E-04
*Lpar1, Sox10, Lgi4, Lamb1, Erbb3, App, Srgap2, Cd9, Adgrg1, Metrn, Mag, Cnp, S100b*		
Glial cell differentiation (GO:0010001)	7.44	2.38E-03
*Lpar1, Sox10, Lgi4, Erbb3, App, Cd9, Metrn, Mag, Cnp, S100b*		
Regulation of supramolecular fiber organization (GO:1902903)	4.84	2.53E-03
*Capg, Lpar1, Nav3, Mid1ip1, Arl2, Chadl, Tmsb10, Cryab, Arpc1a, App, Limch1, Sorbs3, Emilin1, S100a10*		
Extracellular structure organization (GO:0043062)	3.87	4.98E-02
*Lamb1, App, Col16a1, Itga6, Itgb8, Vwa1, Emilin1, Col6a1, Jam3, Col9a3, Ddr1*		
Biological adhesion (GO:0022610)	3.19	1.60E-03
*Lrrc4b, Cd81, Lamb1, Il1rap, Gldn, App, Col16a1, Mpz, Hepacam, Itga6, Srgap2, Clic1, Itgb8, Ttyh1, Ctnna3, Cd9, Sorbs3, Adgrg1, Emilin1, Mag, Cnn3, Col6a1, Jam3, Ddr1*		
Cell adhesion (GO:0007155)	3.07	2.72E-03
*Lrrc4b, Lamb1, Il1rap, Gldn, App, Col16a1, Mpz, Hepacam, Itga6, Srgap2, Clic1, Itgb8, Ttyh1, Ctnna3, Cd9, Sorbs3, Adgrg1, Emilin1, Mag, Cnn3, Col6a1, Jam3, Ddr1*		
Neuron development (GO:0048666)	2.9	2.82E-02
*Sema3b, Lgi4, Lamb1, Ahi1, Gldn, Gas7, App, Ngfr, Rps6ka5, Srgap2, Rab13, Ank3, Mag, Cnp, S100b, Jam3, Mtmr2, Dclk1, Sh3kbp1, Ddr1*		
Regulation of cellular component movement (GO:0051270)	2.79	1.48E-02
*Sema3b, Lpar1, Cd81, Nav3, Tmsb10, Lamb1, Srgap2, Erbb3, App, Itga6, Srgap2, Limch1, Rassf4, Ctnna3, Cd9, Adgrg1, Emilin1, Tgfbr3, Jam3, Snai2, Gpsm3, Mal*		
Cytoskeleton organization (GO:0007010)	2.65	1.81E-02
*Capg, Tacc2, Arl2, Tmsb10, Cryab, Epb41l2, Arpc1a, Mical3, Arhgap26, Shroom2, Gas7, Ccser2, Marcks, Srgap2, Limch1, Rab13, Sorbs3, Tacc1, Ank3, Cnn3, Cnp, Diaph3, Sh3kbp1, Tubb2a*		
Plasma membrane bounded cell projection organization (GO:0120036)	2.5	4.96E-02
*Sema3b, Lpar1, Lamb1, Ahi1, Gldn, Gas7, App, Itga6, Ngfr, Rps6ka5, Srgap2, Ttyh1, Rab13, Ank3, Mag, Cnp, S100b, Ehd4, Jam3, Mtmr2, Dclk1, Sh3kbp1, Ddr1, Pmp22*		

### SOX10-regulated TSSs are associated with higher SOX10 ChIP-Seq signal and motif conservation

**Fig. 4:**
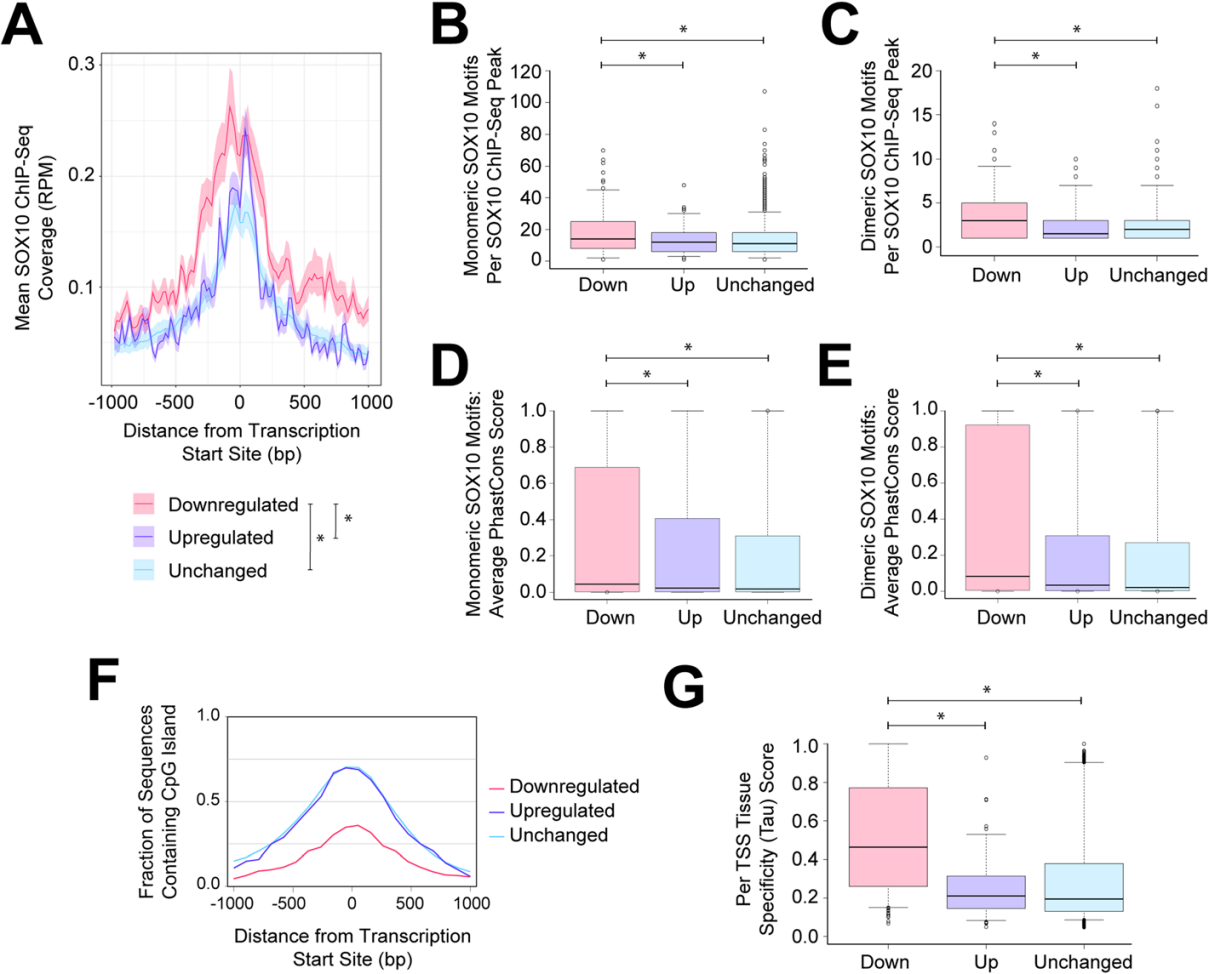
SOX10-dependent transcription start sites are associated with high-affinity SOX10 binding at conserved motifs and restricted expression profiles. **a** Aggregate SOX10 ChIP-Seq data in the 2-kilobase region surrounding TSSs that were downregulated, upregulated, or unchanged in ΔSOX10 S16 cells as in Fig. 3a. X-axis: genomic distance from the TSS (base pairs, bp). Y-axis: average SOX10 ChIP-Seq signal (RPM, reads per million). Asterisk indicates *p* < 0.001. **b** and **c** Monomeric (**b**) and dimeric (**c**) SOX10 binding motifs underlying SOX10 ChIP-Seq peaks associated with each class of TSS. Y-axis: distribution of number of motifs per peak. **d** and **e** Conservation scores of monomeric (**d**) and dimeric (**e**) SOX10 binding motifs as defined in panels **b** and **c**. Y-axis: distribution of conservation scores. **b**-**e** Whiskers extend to the 5th and 95th percentile of the data. Asterisks indicate *p* < 0.01. **f** CpG islands surrounding the downregulated, upregulated, and unchanged TSSs from the ΔSOX10 S16 model. X-axis: distance from the TSS (bp, base pairs). Y-axis: fraction of regions harboring a CpG island, calculated in 100 bp bins. **g** Tau scores for TSSs that were downregulated, upregulated, and unchanged in the ΔSOX10 S16 model, measured across 11 mouse tissues. Y-axis: distribution of Tau scores. Whiskers extend to the 5th and 95th percentile of data. Asterisk, p < 0.001

As described above, it is notable that only 265 TSSs (5%) exhibit the expected downregulation upon loss of SOX10 in vitro, so we sought features which distinguish TSSs that are SOX10 dependent from the other two classes. We first asked if there are differences in the SOX10 ChIP-Seq signal at TSSs within each class. We performed a metagene analysis using sciatic nerve SOX10 ChIP-Seq data [19, 30], this time assessing the aggregate signal in a 2-kilobase window surrounding TSSs that were downregulated (265 TSSs), upregulated (100 TSSs), or unchanged (3766 TSSs) in the ΔSOX10 S16 model. This revealed that SOX10-dependent TSSs had higher SOX10 ChIP-Seq signal, consistent with tighter binding and/or increased occupancy compared to upregulated or unchanged TSSs (Fig. 4a). Notably, we established that technical biases related to GC content are unlikely to have confounded this analysis, as SOX10-dependent TSSs do not exhibit a bias toward lower GC content and the SOX10 ChIP-Seq profiles associated with SOX10-dependent TSSs exhibit greater intensity even after binning TSSs for GC content (Supplementary Figures. 3 and 4).

To investigate the mechanism of increased SOX10 ChIP-Seq signal at SOX10-dependent TSSs, we computationally tested for differences in the presence and/or composition of SOX10 binding motifs associated with these TSSs. Using an inclusive algorithm that allowed a one-base mismatch from a high-confidence SOX10 binding motif [19, 42], the DNA sequence underlying each SOX10 ChIP-Seq peak was assessed for predicted SOX10 binding sites. SOX10 ChIP-Seq peaks associated with SOX10-dependent TSSs exhibited an increase in the number of SOX10 binding motifs compared to those associated with upregulated or unchanged TSSs; this was the case for both monomeric (Fig. 4b) or dimeric (Fig. 4c) binding motifs. However, we found no differences in the sequence composition of the motifs associated with each group (data not shown).

Evolutionary conservation can predict the functional significance of non-coding gene regulatory sequences [43]. We analyzed the conservation of the SOX10 binding motifs identified above using phastCons evolutionary conservation scores [44] across 13 vertebrate species. This revealed that SOX10 binding motifs at SOX10-dependent TSSs have higher conservation scores compared to those at TSSs that were not dependent on SOX10 for expression (Fig. 4d and e). Importantly, this shift toward higher sequence conservation supports the idea that these motifs carry greater functional importance and supports the utility of sequence conservation-based prioritization of SOX10 binding motifs [23–25]. As a whole, the increased SOX10 ChIP-Seq signal, increased number of SOX10 binding motifs, and higher conservation scores for motifs associated with SOX10-dependent TSSs are consistent with the regulation of these 265 TSSs by SOX10-responsive promoters.

### SOX10-regulated TSSs are associated with characteristics of cell type-specific regulation

To further explore differences between TSSs that are downregulated (265), upregulated [45], or unchanged (3766) upon SOX10 deletion, we assessed other features of promoter elements. Algorithm-based sequence analysis revealed no enrichment for TATA box [46] or initiator motifs [47] at SOX10-dependent TSSs (data not shown), and the genomic regions surrounding these TSSs exhibit no difference in GC content (Supplementary Figure 3). However, the 265 SOX10-dependent TSSs are depleted of CpG islands compared to the other groups of TSSs; 35% of the 265 SOX10-dependent TSSs reside within a CpG island, while 70% of the TSSs from other classes are associated with a CpG island (Fig. 4f). CpG islands are enriched at the promoters of ubiquitously expressed transcripts [48]. Thus, the depletion of CpG islands at SOX10-dependent promoters suggests that the associated TSSs are enriched for restricted expression patterns. To address this, we sought to analyze the expression of TSSs that were downregulated, upregulated, or unchanged in the ΔSOX10 S16 cells across a multitude of tissues using previously generated datasets. Importantly, we considered that traditional RNA-Seq library preparations are known to exhibit reduced coverage at 5′ ends, complicating the interpretation of results especially at lowly-expressed loci. Therefore, we chose instead to perform these analyses by assessing TSS expression across 11 mouse tissues using publicly available cap analysis of gene expression (CAGE) data [49]. Each TSS was assigned a tissue specificity (Tau) score [50] based on the breadth and strength of expression, where 0 indicates ubiquitous expression and 1 indicates highly restricted expression. Interestingly, the 265 SOX10-dependent TSSs exhibit greater Tau scores compared to TSSs that were upregulated and unchanged upon loss of SOX10 (Fig. 4g), indicating more restricted expression patterns for the SOX10-dependent TSSs. These analyses also revealed that the expression of SOX10-dependent TSSs is generally highest in the spinal cord, skin, and cortex (Supplementary Figure 5); each of these tissues harbor SOX10-positive cells including oligodendrocytes, Schwann cells, and melanocytes. These data warrant further study of the associated gene products to provide insights into the biology of Schwann cells and other SOX10-positive cell types.

### Developmental and isoform-specific targets of SOX10 in Schwann cells

We identified transcripts that may play important roles in PNS myelination based on: (***i***) expression and association with SOX10-bound promoter marks in vivo; (***ii***) expression in differentiating primary Schwann cells in vitro; and (***iii***) SOX10-dependent expression in a SOX10 knockout model. As SOX10 is known to induce the expression of developmentally-regulated genes in Schwann cells, we asked to what extent SOX10-dependent TSSs in the S16 model exhibit regulated expression during primary Schwann cell differentiation. A comparison of these datasets revealed that of the 265 TSSs expressed in sciatic nerve and downregulated in the ΔSOX10 S16 model, 132 (50%) were upregulated upon primary cell differentiation, supporting that these SOX10-regulated transcripts are relevant for myelinating cells. Moreover, 24 SOX10-dependent TSSs (9%) were downregulated with differentiation, indicating that these SOX10 targets may function during earlier developmental stages. Finally, 87 SOX10-dependent TSSs (33%) were unchanged during differentiation, suggesting that these SOX10 target transcripts may be important at multiple stages of Schwann cell development (Supplementary Table 1).

Compared with conventional expression studies (e.g., bulk RNA-Seq), our TSS-focused dataset permits a detailed exploration of the isoform diversity of gene expression. Therefore, we next assessed the complexity of annotated transcripts in the human RefSeq database for each of the loci containing SOX10-dependent TSSs. The 265 SOX10-dependent TSSs identified in this analysis map to 169 unique gene loci, and 76 of these loci (45%) are annotated with multiple TSSs in the human RefSeq database (Supplementary Table 2). At 55 loci (32%) the alternative TSSs confer unique protein-coding sequences to the resulting transcripts (Supplementary Table 2). Therefore, the identification of SOX10-regulated TSSs at these loci provides insight into specific transcript and protein isoforms that play important roles in Schwann cell function.

### Validation of SOX10-regulated promoters and gene isoforms at four previously unreported target loci

Our studies provide the first comprehensive assessment of TSS use in Schwann cells. To confirm that we identified bona-fide SOX10-regulated promoters and to illustrate biological insights provided by our TSS-specific gene expression studies, we performed a series of functional studies to validate four previously unreported SOX10 target loci that were selected based on the novelty of the findings and/or the possible relevance to Schwann cell biology.

#### Actin related protein 2/3 complex subunit 1A (ARPC1A)

**Fig. 5:**
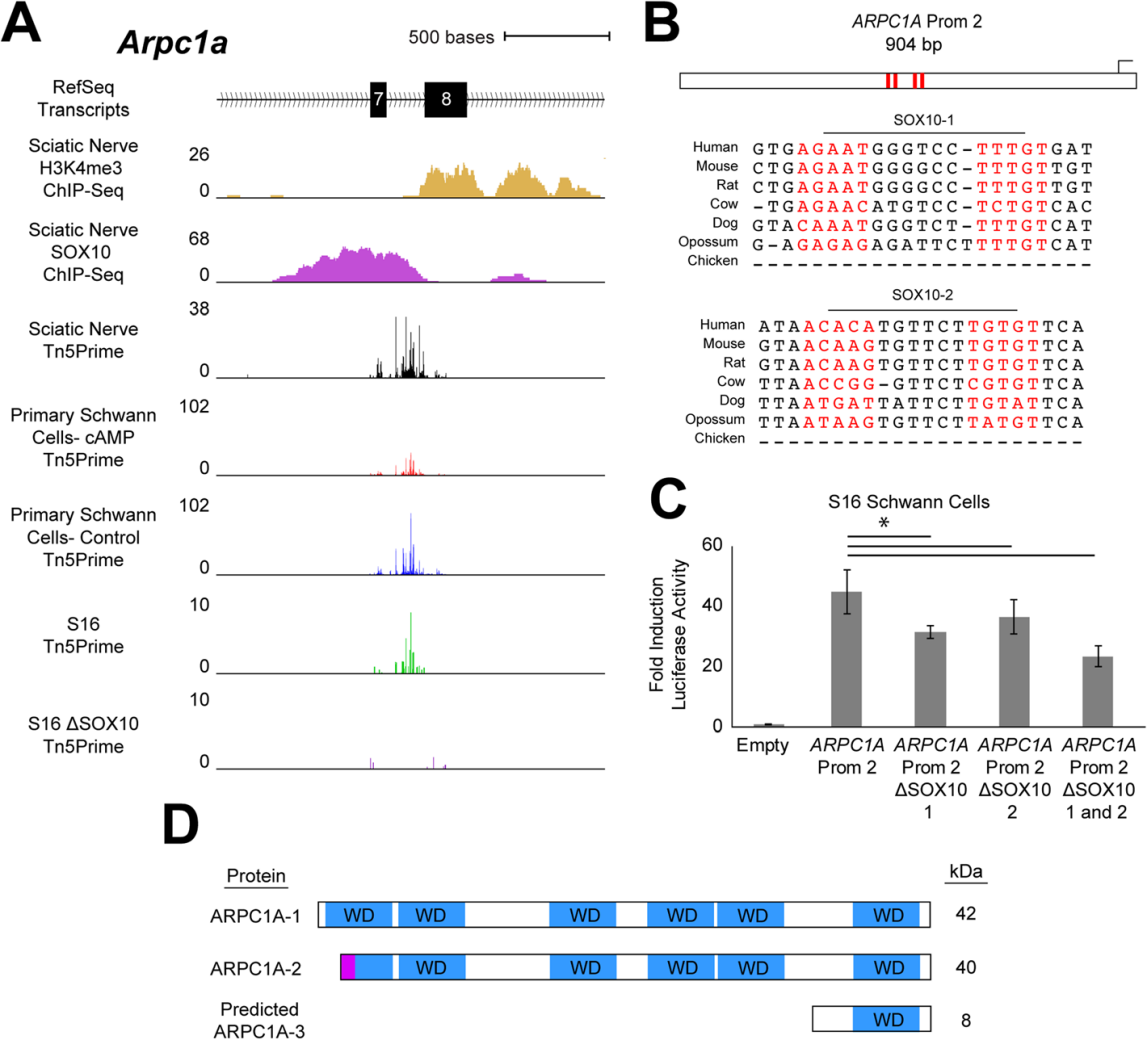
SOX10 regulates the expression of a novel transcription start site at *Arpc1a*. **a** The genomic region surrounding exons 7 and 8 of the rat *Arpc1a* locus. Y-axes for H3K4me3 and SOX10 ChIP-Seq data: fold enrichment of sequencing reads above chromatin input. Y-axes for Tn5Prime data from rat sciatic nerve, CPT-cAMP- (cAMP) and vehicle-treated (Control) primary Schwann cells, and unmodified and ΔSOX10 S16 cells: number of transcript 5’ends mapped per base, in reads per million. **b** The 904-base pair *ARPC1A* Prom 2 is shown along with the position of the two SOX10 dimeric consensus sequences (red bars and red text). The seven species utilized for comparative sequence analysis are shown on the left. **c***ARPC1A* Prom 2 (with or without the dimeric SOX10 sequences, as indicated) was tested in luciferase reporter assays in cultured Schwann (S16) cells. Y-axis: fold induction of luciferase activity; error bars indicate standard deviations. Asterisk indicates p < 0.05. **d** Annotated ARPC1A protein isoforms 1 and 2 contain WD40 repeats (WD, blue) and isoform-specific N-terminal sequences (purple). A cartoon of the predicted protein product of the SOX10-regulated *Arpc1a* transcript (isoform 3) is presented at the bottom of the panel. Predicted molecular weights based on amino acid sequences are shown in kilodaltons (kDa) on the right

The *ARPC1A* locus is annotated with a single TSS, with downstream alternative splicing regulating the expression of two annotated transcripts (Supplementary Figure 6A). However, our studies identified a previously unannotated TSS in the seventh intron of the rat *Arpc1a* locus (Fig. 5a). Furthermore, this TSS is utilized in sciatic nerve at a high rate (detected at 273 counts per million per 100 bases) relative to the upstream, annotated TSS (detected at 155 counts per million per 100 bases). The expression of this intronic TSS: (***i***) is associated with a SOX10-bound promoter in sciatic nerve; (***ii***) exhibits a 50% downregulation upon differentiation in primary Schwann cells (FDR-corrected *p*-value = 4.20 × 10^− 20^); and (***iii***) is largely abolished upon ablation of SOX10 in S16 cells (FDR-corrected *p*-value = 1.18 × 10^− 43^) (Fig. 5a). These data support the identification of a novel TSS and SOX10-regulated promoter at *Arpc1a*.

Sequence analysis identified two dimeric SOX10 binding motifs less than 500 bases upstream of the newly identified TSS (SOX10 Motif-1 and SOX10 Motif-2 in Fig. 5b), which are variably conserved among mammals (Fig. 5b). We used a luciferase reporter assay to test the regulatory activity of a 904 base pair fragment upstream of the TSS (referred to as *ARPC1A* Prom 2) from the orthologous region of the human genome (Supplementary Table 4). In S16 cells, *ARPC1A* Prom 2 exhibits a 45-fold induction of luciferase activity relative to an empty control vector (Fig. 5c; p-value = 9 _× 10^− 11^), consistent with strong regulatory activity. We mutagenized Prom 2 to delete each SOX10 motif in isolation and together. Deletion of SOX10 Motif-1 reduced the activity of the element by ~ 30% (Fig. 5c; p-value = 1.9 × 10^− 4^) while deletion of Motif-2 reduced activity by ~ 20% (Fig. 5c; p-value = 0.02). Consistent with an additive effect, deletion of both motifs reduced the activity of *ARPC1A* Prom 2 by ~ 50% compared to the wild-type element (Fig. 5c; p-value = 2.7 × 10^− 6^). Taken together these results support the validation of *ARPC1A* Prom 2 as an active, SOX10-responsive regulatory element in Schwann cells.

To understand the gene products generated from *ARPC1A* Prom 2, we characterized the *Arpc1a* transcript expressed from Prom 2 via RT-PCR using cDNA generated from rat sciatic nerve RNA, followed by DNA sequencing. These efforts confirmed that the transcript originating from *Arpc1a* Prom 2 includes exons 1B (comprising annotated exon 8 and upstream intronic sequence), 9, and 10 (Supplementary Figure 6). Based on Kozak sequence assessments, we predict that this transcript directs the expression of a 71 or 74 amino acid protein with a molecular weight of ~ 8 kDa that lacks 5 of the 6 WD40 repeat regions of full-length ARPC1A (Fig. 5d); attempts to validate the expression of a stable ARPC1A protein arising from this transcript with commercially available ARPC1A antibodies were unsuccessful. In sum, our data support the SOX10-regulated expression of a novel *Arpc1a* transcript isoform in Schwann cells that encodes an N-terminally truncated protein isoform.

#### Chimerin 2 (CHN2)

**Fig. 6:**
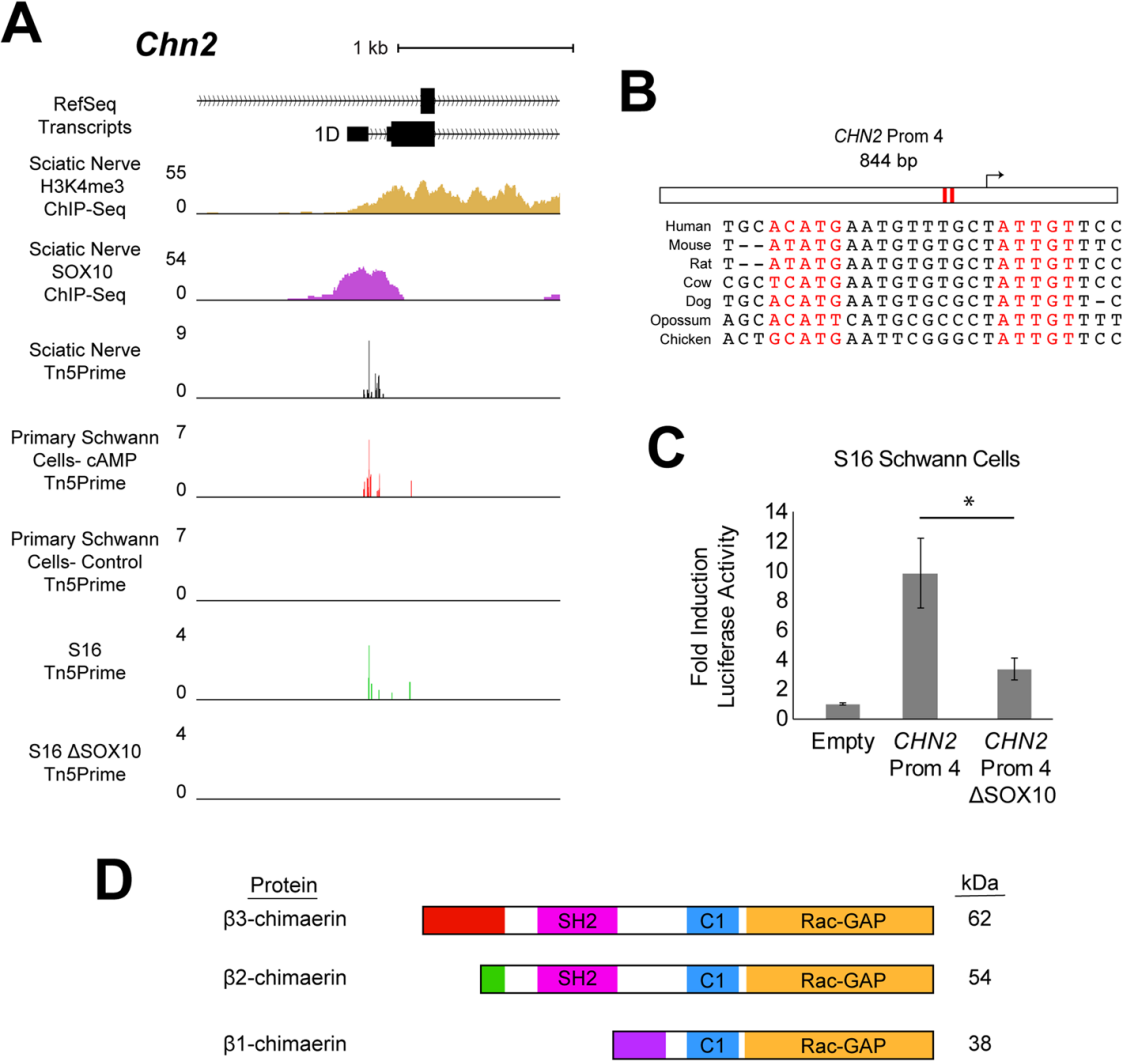
SOX10-dependent expression of *Chn2* transcripts originating at exon 1D. **a** The genomic region surrounding exon 1B at the rat *Chn2* locus. Y-axes for H3K4me3 and SOX10 ChIP-Seq data: fold enrichment of sequencing reads above chromatin input. Y-axes for Tn5Prime data from rat sciatic nerve, CPT-cAMP- (cAMP) and vehicle-treated (Control) primary Schwann cells, and unmodified and ΔSOX10 S16 cells: number of transcript 5’ends mapped per base, in reads per million. **b** The 844-base pair *CHN2* Prom 4 is shown along with the position of the SOX10 dimeric consensus sequence (red bars and red text). The seven species utilized for comparative sequence analysis are shown on the left. **c***CHN2* Prom 2 (with or without the dimeric SOX10 sequence, as indicated) was tested in luciferase reporter assays in cultured Schwann (S16) cells. Y-axis: fold induction of luciferase activity; error bars indicate standard deviations. Asterisk indicates *p* < 1 × 10^− 6^. **d** β-chimaerin isoforms 1, 2, and 3. β2-and β3-chimaerins contain Src-homology 2 (SH2, magenta), diacylglycerol binding (C1, blue), and Rac-GTPase activating (Rac-GAP, orange) domains, and are distinguished by isoform-specific N-terminal sequences (red and green). β1-chimaerin includes an isoform-specific N-terminal sequence (purple). Predicted molecular weights based on amino acid sequences are shown in kilodaltons (kDa) on the right

*CHN2* comprises a complex transcriptional unit with five TSSs and downstream alternative splicing events annotated at the human locus (Supplementary Figure 7A and data not shown). Our sciatic nerve-derived Tn5Prime data at *Chn2* revealed predominant expression from a single TSS at exon 1D (Fig. 6a). The TSS: (***i***) is associated with a SOX10-bound promoter in sciatic nerve; (***ii***) exhibits little expression in untreated primary Schwann cells and is induced 24-fold upon cAMP-mediated differentiation (FDR-corrected *p*-value = 0.000592); and (***iii***) is expressed in unmodified S16 Schwann cells but is lost with deletion of SOX10 (FDR-corrected p-value = 0.0000165) (Fig. 6a). Therefore, the *Chn2* TSS at exon 1D was identified through our integrated analysis as a high confidence, SOX10-dependent transcript that may be relevant for differentiating Schwann cells.

Sequence analysis identified a dimeric SOX10 binding motif that maps ~ 150 bases upstream of the TSS at *Chn2* exon 1D and that is variably conserved among vertebrates (Fig. 6b). To assess the regulatory activity of this element, we tested an orthologous 844 base pair region from the human genome (Supplementary Table 4) in luciferase reporter assays. *CHN2* Prom 4 induced luciferase activity 10-fold greater than the empty control in unmodified S16 cells (Fig. 6c; *p*-value = 5.2 × 10^− 10^), consistent with regulatory activity in this context. Deletion of the dimeric SOX10 motif resulted in 70% reduced activity (Fig. 6c; p-value = 2.9 × 10^− 7^). These findings support an important role for SOX10 and the dimeric motif sequence in mediating the activity of *CHN2* Prom 4 in Schwann cells.

We next sought to validate the identity of the *Chn2* gene products generated by this promoter in Schwann cells in vivo. We performed RT-PCR using cDNA generated from rat sciatic nerve RNA followed by DNA sequencing to identify transcripts arising from *Chn2* Prom 4. These studies revealed that this transcript includes *Chn2* exon 1D followed by all of the downstream protein-coding exons (Supplementary Figure 7) and confirmed that *Chn2* Prom 4 directs expression of transcripts coding for the β1-chimaerin protein isoform in sciatic nerve. Attempts to confirm expression of this protein in Schwann cells using commercially available β-chimaerin antibodies were unsuccessful. In sum, our data support the SOX10-mediated expression of β1-chimaerin in Schwann cells and suggest a role for this protein isoform in mature, myelinating cells.

#### Discoidin domain receptor tyrosine kinase 1 (DDR1)

**Fig. 7:**
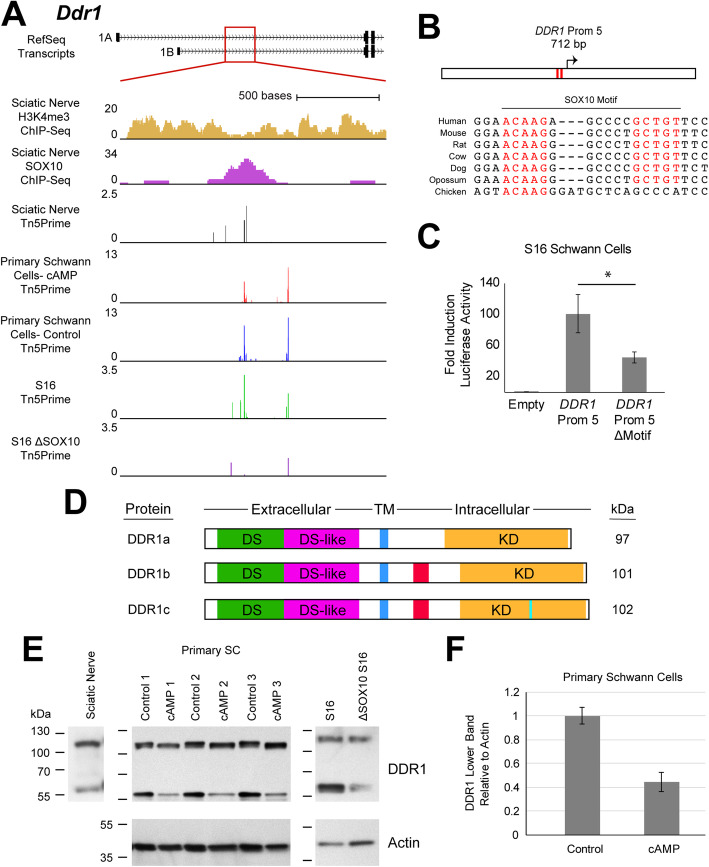
SOX10 regulates the expression of a novel *Ddr1* transcription start site in Schwann cells. **a** The genomic locus at rat *Ddr1* intron 1. Y-axes for H3K4me3 and SOX10 ChIP-Seq data: fold enrichment of sequencing reads above chromatin input. Y-axes for Tn5Prime data from rat sciatic nerve, CPT-cAMP- (cAMP) and vehicle-treated (Control) primary Schwann cells, and unmodified and ΔSOX10 S16 cells: number of transcript 5’ends mapped per base, in reads per million. **b** The 712-base pair *DDR1* Prom 5 is shown along with the position of the SOX10 dimeric consensus sequence (red bars and red text). The seven species utilized for comparative sequence analysis are shown on the left. **c***DDR1* Prom 5 (with or without the dimeric SOX10 sequence, as indicated) was tested in luciferase reporter assays in cultured Schwann (S16) cells. Y-axis: fold induction of luciferase activity; error bars indicate standard deviations. Asterisk indicates p < 1 × 10^− 4^. **d** DDR1 isoforms a, b, and c contain discoidin (DS, green), discoidin-like (DS-like, magenta), transmembrane (TM, blue) and kinase (KD, orange) domains, and are distinguished by insertions in the intracellular sequences (red and light blue). Predicted molecular weights based on amino acid sequences are shown in kilodaltons (kDa) on the right. **e** DDR1 protein expression in sciatic nerve, primary Schwann cells, and S16 cells. Actin was used as a protein loading control. Numbered dashes to the left of each blot indicate the position of protein size markers in kilodaltons (kDa). **f** The intensity of the lower DDR1 band relative to Actin signal in control and cAMP-treated primary Schwann cells. The average across three independent samples is indicated by the bar height. Error bars indicate standard deviations

Our Tn5Prime data from rat sciatic nerve identified a previously unannotated TSS in the first intron of the *Ddr1* locus (Fig. 7a). The expression of this TSS: (***i***) is associated with a SOX10-bound promoter in sciatic nerve; (***ii***) exhibits no change upon differentiation in primary Schwann cells; and (***iii***) is dependent on SOX10 in our S16 model, with > 90% reduced expression in ΔSOX10 S16 cells compared to control cells (FDR-corrected *p*-value = 0.027) (Fig. 7a). These data support the identification of an unannotated, SOX10-dependent TSS at *Ddr1* that is utilized in Schwann cells and may be relevant across multiple stages of Schwann cell development.

Sequence analysis identified a dimeric SOX10 binding motif located ~ 100 bases upstream of the SOX10-dependent TSS; this dimeric motif is perfectly conserved among mammals and one of the monomers is conserved among vertebrates (Fig. 7b). To assess the activity of the promoter associated with the novel TSS, we tested a 712 base pair orthologous fragment from the human genome, referred to as *DDR1* Prom 5 (Supplementary Table 4), in luciferase reporter assays. *DDR1* Prom 5 exhibited high regulatory activity in S16 cells with 100-fold induction of luciferase activity relative to the empty control vector (Fig. 7c; p-value = 2 × 10^− 8^). Deletion of the dimeric SOX10 motif resulted in a 60% reduction in activity (Fig. 7c; p-value = 2 × 10^− 5^). These data validate the regulatory activity of the promoter and identify a dimeric SOX10 binding motif directly upstream of the TSS that is required for a large portion of the promoter activity.

To understand the functional implications of the SOX10-regulated promoter at *Ddr1*, we characterized the *Ddr1* transcript and protein products arising from the Tn5Prime-defined TSS. To identify transcripts arising from this promoter, we performed an RT-PCR with cDNA generated from rat sciatic nerve RNA and sequenced the resulting amplicons. These studies confirmed the presence of full-length *Ddr1* transcripts that originate at the Tn5Prime-defined TSS (exon 1E; note that the rat locus is annotated with two TSSs upstream of exon 1E, as shown in Fig. 7a, while the human locus is annotated with four upstream TSSs corresponding to exons 1A-1D, not shown) and include or exclude *Ddr1* exon 12 (Supplementary Figure 8). These transcripts encode the two most abundant DDR1 protein isoforms, DDR1a (encoded by exclusion of exon 12) and DDR1b (encoded by inclusion of exon 12) (Fig. 7d).

We next assessed the expression of DDR1 protein products in Schwann cells via western blot analyses. Based on our Tn5Prime data, we anticipated the expression of DDR1 protein products in Schwann cells that do and do not arise from the SOX10-regulated TSS at exon 1E. To confirm DDR1 expression in Schwann cells we analyzed protein lysates from: rat sciatic nerve; untreated and cAMP-treated primary Schwann cells; and unmodified and ΔSOX10 S16 cells. Indeed, this revealed the expression of protein product(s) with an apparent size of ~ 120 kDa in each model, consistent with the expression of full-length DDR1 isoforms in Schwann cells (Fig. 7e). A smaller fragment of approximately 60 kDa was also detected in each model (Fig. 7e). This is consistent with previous reports describing metalloproteinase-mediated shedding of the DDR1 extracellular domain, leaving a ~ 62 kDa membrane-bound intracellular fragment [51–53]. This finding for the first time suggests that ectodomain cleavage regulates DDR1 signaling in the context of the peripheral nerve. Interestingly, there is a slight but consistent shift in the size of full-length DDR1 between control- and cAMP-treated primary Schwann cells, with an apparently smaller protein detected in cAMP-treated cells compared to controls. Moreover, cAMP-treated primary Schwann cells exhibit decreased signal corresponding to the cleaved DDR1 fragment (Fig. 7e-f, p-value = 8.7 × 10^− 4^). Overall, our findings support the conclusion that SOX10 induces the expression of DDR1 in Schwann cells and suggest that DDR1 protein expression and function may be subject to complex regulatory processes in peripheral nerve.

#### Growth arrest specific 7 (GAS7)

**Fig. 8:**
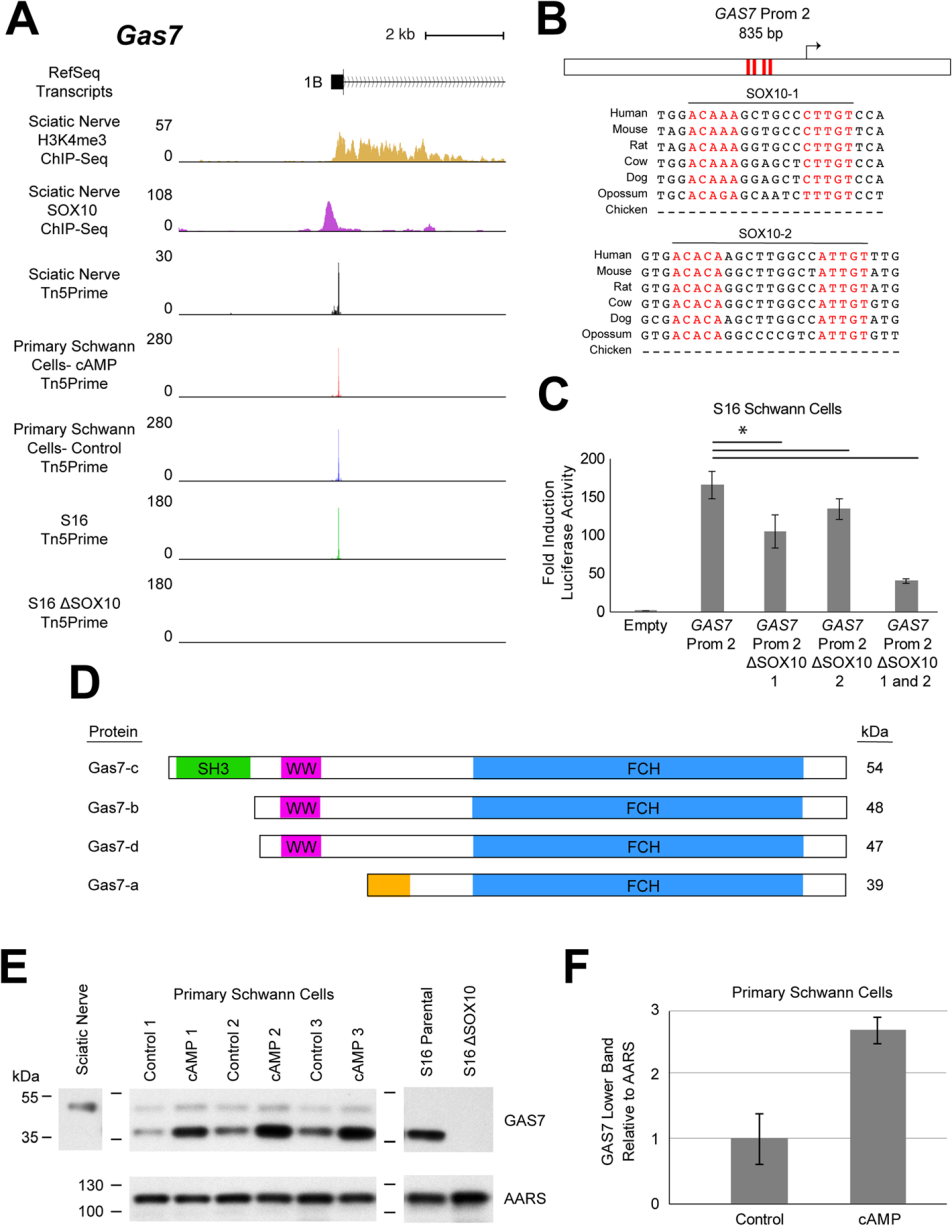
SOX10-dependent expression of a *Gas7* transcription start site at exon 1B. **a** The genomic region surrounding *GAS7* exon 1B at the rat *Gas7* locus. Y-axes for H3K4me3 and SOX10 ChIP-Seq data: fold enrichment of sequencing reads above chromatin input. Y-axes for Tn5Prime data from rat sciatic nerve, CPT-cAMP- (cAMP) and vehicle-treated (Control) primary Schwann cells, and unmodified and ΔSOX10 S16 cells: number of transcript 5’ends mapped per base, in reads per million. **b** The 835-base pair *GAS7* Prom 2 is shown along with the position of the SOX10 dimeric consensus sequences (red bars and red text). The seven species utilized for comparative sequence analysis are shown on the left. **c***GAS7* Prom 2 (with or without the dimeric SOX10 sequences, as indicated) was tested in luciferase reporter assays in cultured Schwann (S16) cells. Y-axis: fold induction of luciferase activity; error bars indicate standard deviations. Asterisk indicates *p* < 0.005. **d** GAS7 isoforms a, b, c, and d. Isoform Gas7-c contains Src homology 3 (SH3, green), WW (magenta), and Fes/Cip4 homology (FCH, blue) domains. Gas7-a contains an isoform-specific N-terminal sequence (gold). Predicted molecular weights based on amino acid sequences are shown in kilodaltons (kDa) on the right. **e** GAS7 protein expression in sciatic nerve, primary Schwann cells, and S16 cells. AARS was used as a protein loading control. Numbered dashes to the left of each blot indicate the position of protein size markers in kilodaltons (kDa). **f** The intensity of the lower GAS7 band relative to AARS signal in control and cAMP-treated primary Schwann cells. The average across three independent samples is indicated by the bar height. Error bars indicate standard deviation

The human *GAS7* locus harbors four annotated TSSs (Supplementary Figure 9A) that encode distinct protein isoforms. Our Tn5Prime data from rat sciatic nerve identified a single predominant TSS at the *Gas7* locus that maps to exon 1B (Fig. 8a). This TSS: (***i***) maps to H3K4me3 and SOX10 ChIP-Seq peaks; (***ii***) is expressed similarly in control and cAMP-treated primary Schwann cells; (***iii***) and exhibits expression in S16 cells that is largely lost upon deletion of SOX10 (Fig. 8a; FDR-adjusted p-value = 2.15 × 10^− 165^). In sum, our data support the identification of a candidate SOX10-regulated promoter and associated TSS at *Gas7* in Schwann cells.

Sequence analysis identified two dimeric binding motifs (SOX10 Motif-1 and SOX10 Motif-2 in Fig. 8b) that are separated by ~ 30 base pairs and reside less than 350 bases upstream of the Tn5Prime-defined TSS; these motifs are highly conserved among mammals (Fig. 8b). To assess the regulatory activity of the promoter region associated with the above TSS, an 835 base pair fragment containing the orthologous sequence from human (Supplementary Table 4) was tested in luciferase reporter assays. This promoter—referred to as *GAS7* Prom 2—induces > 160-fold luciferase activity relative to an empty control vector (Fig. 8c; *p*-value = 3 × 10^− 13^), consistent with strong regulatory activity. To test the contributions of SOX10 Motif-1 and Motif-2 to the regulatory activity, each motif was deleted individually; this resulted in 30% (p-value = 2 × 10^− 5^) and 20% (p-value = 1 × 10^− 3^) reduced activity relative to the wild-type construct, respectively (Fig. 8c). When both dimeric motifs were deleted together, the activity of *GAS7* Prom 2 was reduced by approximately 75% (Fig. 8c; p-value = 2 × 10^− 11^), indicating that these sequences are required for a large portion of promoter activity in this assay. These studies support the identification of an exon 1B-associated TSS at *Gas7* that is proximally regulated by SOX10 via two dimeric binding motifs.

To determine the relevance of the SOX10-regulated promoter and to identify the *Gas7* gene products that are expressed in Schwann cells, we characterized transcripts and proteins originating from *Gas7* Prom 2 in peripheral nerve. We performed an RT-PCR from sciatic nerve cDNA to amplify transcripts derived from this promoter and subjected the amplicons to DNA sequencing. This confirmed expression of a full-length, exon 1B-derived *Gas7* transcript in sciatic nerve (Supplementary Figure 9) that encodes the Gas7-b protein isoform (Fig. 8d). We next assessed GAS7 protein expression in Schwann cells via western blot analyses. Our Tn5Prime data support the predominant, if not exclusive, utilization of the exon 1B-associated *Gas7* TSS in each cellular model (Supplementary Figure 10). Therefore, we anticipate the expression of GAS7 protein products in Schwann cells that arise only from the SOX10-regulated TSS at exon 1B. To confirm GAS7 expression in Schwann cells we analyzed protein lysates from: rat sciatic nerve; untreated and cAMP-treated primary Schwann cells; and unmodified and ΔSOX10 S16 cells. Results from sciatic nerve lysates confirmed the expression of a protein with an apparent size consistent with the GAS7-b isoform (~ 48 kDa) (Fig. 8e). However, analysis of primary Schwann cell lysates indicated the expression of the same GAS7 protein, along with a smaller protein product (Fig. 8e). Moreover, cAMP-treated cells exhibited a consistent, ~ 2.5-fold upregulation of the smaller GAS7 protein relative to control cells (Fig. 8f, p-value = 2.7 × 10^− 3^), suggesting that GAS7 protein expression is dynamically regulated during Schwann cell differentiation. Finally, testing S16 cell lysates revealed that these cells predominantly, if not exclusively, express the smaller GAS7 protein product; this protein is absent in ΔSOX10 S16 cells (Fig. 8e), as expected for a product arising from the SOX10-dependent transcript (Fig. 8a).

The smaller GAS7 protein detected in primary Schwann cells and S16 cells exhibits an apparent size consistent with the GAS7-a protein isoform (~ 39 kDa), despite the fact that our TSS mapping data provide no evidence for transcripts originating at the TSS associated with this isoform (exon 1D; Supplementary Figure 10). To determine if the differential protein expression that we observed (Fig. 8e) results from context-dependent alternative splicing or sequence variation of exon 1B-derived transcripts, we repeated the exon 1B-anchored RT-PCR (Supplementary Figure 9) using RNA from untreated and cAMP-treated primary Schwann cells, and from parental S16 cells, and subjected the resulting amplicons to next-generation sequencing. This confirmed that the transcript sequences derived from exon 1B are identical in each cellular context tested (data not shown), suggesting that post-transcriptional and/or translational mechanisms may dictate context-dependent expression of GAS7 protein isoforms in Schwann cells.

## Discussion

In this study we integrated TSS mapping with SOX10 binding and promoter marks to identify SOX10-regulated promoter elements in Schwann cells genome-wide. In so doing, we have generated a prioritized list of SOX10-bound, active TSSs that exhibit differentiation-dependent expression patterns and/or SOX10-dependent expression in Schwann cells. This dataset of prioritized genes and transcripts represents a valuable resource for the study of SOX10 and Schwann cell biology. We suggest that functional studies at the loci of interest identified by our efforts (Supplementary Table 1) should be completed to interrogate whether SOX10-bound promoters reflect a functional requirement for these genes in peripheral myelination. Furthermore, where applicable these studies should be designed with isoform-specific biology in mind, as there may be particular physiological relevance associated with SOX10-regulated isoforms. Notably, this may be most relevant for the study of genes that are expressed broadly across many tissues. In these cases SOX10-mediated expression from one TSS may impart transcript and/or protein isoform specificity to Schwann cells that is not well appreciated by traditional, whole gene transcriptomic analyses.

We found that a small portion (5%) of the TSSs bound by SOX10 in vivo exhibited decreased activity upon the loss of SOX10 in vitro. Though seemingly discrepant, this finding is consistent with other studies showing that a small percentage of genes near ChIP-Seq peaks for a given factor exhibit altered expression with modulation of that factor [54–57]. Furthermore, we do not observe upregulation of the functionally related family members SOX8 and SOX9 in this model (data not shown), suggesting that our results cannot be explained by a compensatory mechanism. Thus, it may be that once the gene expression profile of a fully differentiated myelinating cell has been established [as modeled by high myelin gene expression in unmodified S16 cells [37]] it is relatively stable, and though SOX10 binds widely throughout the genome, it may not be required for expression at certain loci. It is notable that the loss of SOX10 in fully mature myelinating Schwann cells in vivo is known to induce demyelination and loss of the differentiated state [13]. Our data show that myelin genes *Mbp*, *Mpz*, and *Pmp22* are among the downregulated genes in ΔSOX10 S16 cells, indicating that misregulated expression of critical myelin loci is sufficient to radically affect the myelinating phenotype of differentiated cells upon loss of SOX10. Furthermore, it is known that SOX10 interacts with many transcription factors and chromatin modifiers to mediate target gene expression in Schwann cells and in other cell types [9–11, 18, 34, 58]; indeed, some of these factors are known to be SOX10 target genes themselves [14, 17]. Therefore, an important consideration in the interpretation of our findings is that there are likely to be indirect effects of SOX10 loss that contribute to the transcriptional profile of SOX10-depleted Schwann cells.

Our genome-wide studies revealed 55 loci harboring a SOX10-dependent TSS that directs the expression of a unique coding sequence. To validate these findings with orthogonal approaches, we performed functional studies at four of these 55 loci using assessments of conservation, luciferase assays, transcript analysis, and (where possible) protein expression assays. We identified a previously unannotated TSS in the seventh intron of *Arpc1a*. The *ARPC1A* locus encodes subunit 1A of the actin related protein 2/3 (Arp2/3) complex, which is critical for the polymerization, organization, and recycling of the actin cytoskeleton [59]. The transcript described here is predicted to encode a protein that lacks a majority of the sequence of full-length ARPC1A isoforms (Fig. 5d). ARPC1 adopts a 7-bladed β-propeller conformation and makes multiple contacts with other subunits in the complex [60]. For this reason, it is unlikely that the protein isoform we predict is capable of assembling into the Arp2/3 complex. However, a region of the ARPC1 protein near the C-terminus extends away from the β-propeller formation and adopts an alpha-helical structure that is likely solvent-accessible [61]. Moreover, this region is thought to interact with factors that activate the Arp2/3 complex and is encoded by the transcript we describe here. Therefore, if this protein isoform is expressed and interacts with stimulating factors, it could perform a repressive function by sequestering those factors away from intact Arp2/3 complexes. Further study is required to test these and other hypotheses about the ARPC1A protein predicted by our experiments.

We identified a SOX10-regulated promoter element at the *Chn2* locus that is associated with expression of the β1-chimaerin protein isoform. The β-chimaerin proteins localize to the cell membrane and function as GTPase activating proteins (GAPs) with specificity for the Rho family small GTPase Rac1 [62]. The membrane localization of β-chimaerin is contingent upon activation by the lipid second messenger diacylglycerol (DAG); thus, β-chimaerin activity is induced via signaling cues from cell surface receptors and activation of phospholipase C [63]. Rac1, in turn, operates in a wide array of cellular functions including cell cycle progression, cytoskeleton regulation, and cellular motility [64]. Importantly, Rac1 is known to be required at multiple stages of Schwann cell development [65–69]; therefore, as negative regulators of Rac1, the β-chimaerin proteins are plausible candidates for roles in Schwann cell biology. Moreover, previous studies established that β1-chimaerin exhibits isoform-specific functional characteristics due to the lack of the N-terminal SH2 domain that acts as an auto-inhibitory module in the β2- and β3-chimaerin isoforms (Fig. 6d) [70]. Indeed, β1-chimaerin localizes to the cellular membrane in response to a 60-fold lower dose of DAG mimic than that required for β2-chimaerin [70]. With the caveat that β1-chimaerin protein expression remains to be confirmed in Schwann cells, our findings and previous reports suggest that SOX10-mediated expression of β1-chimaerin may reflect a requirement for carefully titrated Rac1 activity in developing Schwann cells. Indeed, it has been proposed that precise, temporally regulated levels of Rac1 activity have profound implications for migration and lamellae formation by developing Schwann cells [68]. Further studies will be required to elucidate if the expression of β1-chimaerin, as a potent Rac1 inhibitor, modulates Schwann cell development and function.

We identified a previously unreported, SOX10-regulated intronic TSS in Schwann cells that directs the expression of DDR1 protein isoforms a and b. The *DDR1* locus encodes the discoidin domain receptor tyrosine kinase 1 proteins, members of the DDR family of collagen-activated receptor tyrosine kinases [71, 72]. DDR1 is activated by collagen type IV in addition to other collagen species [71, 73] and has been implicated in cellular functions related to adhesion, migration, and extracellular matrix dynamics [51, 74]. Importantly, collagens are components of the PNS extracellular matrix and play critical structural and signaling roles in Schwann cell development and function [75, 76]. Further, type IV collagen is a constituent of the Schwann cell-deposited basal lamina and is required for proper myelination [77]. Thus, while DDR1 has no known role in Schwann cells, the ability of this protein to act as a collagen type IV receptor makes it a plausible candidate to contribute to Schwann cell-basal lamina interactions. Our findings regarding the differential DDR1 protein products expressed in cAMP-treated primary Schwann cells, which are likely derived from the SOX10-regulated promoter we described here as well as other TSSs at the locus, are suggestive of developmentally regulated processes altering the splicing, post-translational modifications, and/or cleavage dynamics of DDR1 in Schwann cells, and warrant further study. Moreover, in light of our data supporting the use of multiple *Ddr1* TSSs in Schwann cells, it is noteworthy that the distinguishing characteristic of transcripts arising from the SOX10-regulated promoter is not a change to the protein coding sequence but the presence of a unique 5’UTR. In this way, *DDR1* is representative of an interesting class of findings from TSS-centered studies: a novel transcript species that does not impart a protein sequence change. Importantly, 5’UTR sequence variation is known to affect transcript targeting, stability, and translation efficiency [78]; therefore, the use of this alternative promoter may have important implications for the post-transcriptional regulation of *DDR1* in Schwann cells. Importantly, DDR1 is expressed in other SOX10-positive cell types, including oligodendrocytes, melanocytes, and supporting cells of the organ of Corti in the inner ear [79–81], suggesting that SOX10 also mediates DDR1 expression in these cell types via the promoter described here. In melanocytes and supporting cells of the organ of Corti, DDR1 functions in cellular adhesion to basement membranes [80, 81]. This supports the idea that SOX10-mediated expression of DDR1 contributes to basal lamina adhesion and that there may be a similar function for DDR1 in Schwann cells. We propose that DDR1 null mice [82] should be closely examined for peripheral myelination deficits to assess whether DDR1 expression is indeed important for Schwann cells.

The *Gas7* locus encodes growth arrest specific 7, a cytoskeleton regulator protein that localizes to the cellular membrane and functions in actin assembly, microtubule bundling, and membrane outgrowth in vitro [83, 84]. GAS7 has been studied predominantly in neurons, where it mediates the formation of filopodia and lamellipodia [85–87]. While GAS7 has no known role in Schwann cells, cytoskeleton dynamics and the formation of lamellipodia-like cellular extensions by developing Schwann cells are critical for the early stages of PNS myelination [68]. Moreover, a mouse model with reduced expression of GAS7 exhibits behavioral motor dysfunction and motor neuron loss upon aging, consistent with a role for this protein in PNS function [88]. Our studies identified a SOX10-regulated TSS at *Gas7* that encodes GAS7 protein isoform b but that is associated with the expression of multiple GAS7 protein products in Schwann cells. Previous studies showed that neuronal cells transfected with the GAS7-b-encoding cDNA sequence express multiple GAS7 protein isoforms with molecular weights that are consistent with our findings [86, 89]. Like the authors of these previous reports, we propose that leaky ribosomal scanning may mediate the generation of multiple protein products from exon 1B-derived *Gas7* transcripts. Based on the presence of in-frame ATG codons, it is plausible that the smaller protein product is derived from protein translation that initiates at the third in-frame ATG, resulting in a 330 amino acid protein with an expected molecular weight of approximately 39 kDa; this protein is largely similar to the annotated GAS7 protein isoform a (Fig. 8d). In neuronal cells, the expression of GAS7-a has been associated with the induction of lamellipodia formation in vitro [86]. Our finding that the smaller GAS7 protein product is induced by cAMP treatment in primary Schwann cells (Fig. 8e) suggests that as immature Schwann cells differentiate, expression of a GAS7 protein that is similar to GAS7-a mediates the extension of the lamellipodia-like processes that sort and wrap axons [90]. The functional implications of these findings and their relevance to developmental myelination in vivo will require further study, especially given our detection of only the GAS7-b isoform in mature sciatic nerve (Fig. 8e). Nonetheless, our data support the expression of multiple GAS7 protein products in Schwann cells and suggest a role for dynamic and context-dependent expression of these proteins during Schwann cell differentiation.

## Conclusions

We report the first comprehensive assessment of transcription start site (TSS) use in Schwann cells by employing three complementary and biologically relevant models. We validate the identification of bona fide SOX10-regulated promoters at four novel target genes and discuss how these data provide preliminary insights into transcript and protein isoform specificity at these loci. As a whole, our data contribute to the field’s understanding of Schwann cell biology by: (***i***) prioritizing SOX10 response elements and target loci in Schwann cells for further study; (***ii***) providing a functional classification and analysis of SOX10-dependent TSSs; and (***iii***) offering insights into isoform-specific gene expression profiles that will be relevant for myelination and myelin-related disease.

## Methods

### Primary Schwann cell differentiation assay

To study gene expression in a model that estimates myelinating Schwann cell development, we employed cAMP-mediated differentiation of primary Schwann cell cultures [31]. Primary rat Schwann cells (Kerafast, Boston, MA, Catalog Number EMI010) were maintained under standard growth conditions in complete Schwann cell (SC) medium: DMEM plus 10% fetal bovine serum (FBS), 2 mM L-glutamine, 50 U/mL penicillin, 50 g/mL streptomycin, 25 μg/mL gentamicin, 10 nM neuregulin EGF domain, and 2 μM forskolin. Differentiation assays were completed as previously described [31]. Prior to cell plating, culture dishes were treated with 1 mL 0.01% poly-L-lysine solution (Sigma, St. Louis, MO) per 25 cm^2^ of surface area and supplemented with sufficient volume of water to cover the bottom of the dish. Dishes were allowed to sit for 5 min at room temperature, then the solution was aspirated and the chamber was allowed to dry completely. Laminin derived from human fibroblasts (Sigma) was diluted in Hank’s Basic Saline Solution (HBSS; without calcium and magnesium) and applied to dishes at 1 μg laminin per cm^2^. Dishes were incubated for 1 h at room temperature. The laminin solution was removed and dishes were washed with sterile water and allowed to dry completely. On day 1, primary Schwann cells were plated at 2.4 × 10^4^ cells/cm^2^ in complete SC medium. On day 2, medium was removed and replaced with D10 medium: DMEM plus 10% FBS, 2 mM L-glutamine, 50 U/mL penicillin, 50 g/mL streptomycin, and 25 μg/mL gentamicin. On day 3, medium was removed and replaced with D5 medium: DMEM plus 5% FBS, 2 mM L-glutamine, 50 U/mL penicillin, 50 g/mL streptomycin, and 25 μg/mL gentamicin. On day 4, medium was removed and replaced with D5 medium plus 250 μM CPT-cAMP (Axxora, Farmingdale, NY) or vehicle. Subsequently, condition-specific medium was replenished each day. On day 7, cells were washed with HBSS and incubated in 0.15% trypsin to dissociate cells from the dish. Trypsin solution was quenched with D10 medium upon cellular detachment. The resulting cell suspension was centrifuged at 200 x g for 10 min at 4 °C for downstream RNA or protein isolation.

### Generation of S16 ΔSOX10 cell lines

To characterize the role of SOX10 in TSS expression we generated immortalized Schwann cell lines that do not express SOX10. Guide RNAs were designed against the first coding exon at the rat *Sox10* locus, each within 300 bases downstream of the start codon (all primers and oligos acquired from IDT, Coralville, IA; sequences available upon request). Guides were cloned into the PX459 plasmid for co-expression with Cas9 and a puromycin resistance gene and resulting constructs were sequence-verified [91]. S16 cells [36] (ATCC Catalog Number CRL-2941) were grown under standard conditions and plated at 3 × 10^4^ cells/well of a 6-well plate in standard medium: DMEM plus 10% FBS, 2 mM L-glutamine, 50 U/mL penicillin, and 50 g/mL streptomycin. The next day, guide RNA-encoding PX459 plasmids were transfected individually using Lipofectamine 2000 (ThermoFisher Scientific, Waltham, MA) in Opti-Mem (ThermoFisher Scientific) according to manufacturer’s protocol (6 μg DNA/well). Four hours after transfection, solution was removed and replaced with standard growth medium. Twenty-four hours after transfection, cells were treated with growth medium containing 5 μg/mL puromycin. Puromycin-containing medium was replenished the following day. After 48 h of puromycin treatment, cells were returned to standard growth medium. Surviving cells were grown to confluency and a portion of the cells were collected for genomic DNA isolation. Genomic sequences surrounding the guide cut sites were PCR-amplified using PCR Supermix (ThermoFisher Scientific); resulting amplicons were cloned and Sanger sequenced to assess the presence of indels as confirmation of editing activity. Subsequently, edited cell populations were subjected to FACS sorting to isolate single cells in individual wells of 96-well plates for clonal expansion. Resulting clones were subjected to crude DNA isolation using QuickExtract (Epicentre Technologies, Madison, WI) and targeted PCR surrounding the editing site. Amplicons were Tn5-tagmented, barcoded, and sequenced on an Illumina MiSeq sequencer. Clones with exclusively frameshift-bearing alleles (i.e., no detection of unedited alleles or frame-preserving indels) were expanded via standard culture conditions for RNA and protein isolation.

### Protein isolation and western blots

Western blot analysis was used to validate primary Schwann cell differentiation and ΔSOX10 S16 models, as well as locus-specific findings at *Ddr1* and *Gas7*. A single sciatic nerve was submerged in RIPA buffer (Pierce/ThermoFisher Scientific) supplemented with protease inhibitor cocktail (ThermoFisher Scientific) and sonicated. Cells were incubated in 0.15% (primary Schwann cells) or 2.5% (S16 cells) trypsin as described above, collected, and centrifuged at 200 x g for 10 min (primary Schwann cells) or 800 x g for 2 min (S16 cells). Subsequently, medium was removed, cell pellets were washed by resuspending in PBS, then centrifuged again using the same conditions. PBS was removed and cell pellets were resuspended in RIPA buffer (Pierce/ThermoFisher Scientific) supplemented with protease inhibitor cocktail (ThermoFisher Scientific). The resulting lysates were incubated for 30 min at 4 °C then centrifuged at 16,000 x g at 4 °C for 30 min. Lysates were moved into a clean tube and stored at − 20 °C. Protein yield was measured with a BCA Protein Assay (ThermoFisher Scientific). Each 50 μg (sciatic nerve) or 10 μg (primary Schwann and S16 cells) sample of protein was supplemented with 2X SDS sample buffer (ThermoFisher Scientific) and beta-mercaptoethanol, incubated at 99 °C for 5 min, then electrophoresed on a 4–20% gradient Tris-Glycine polyacrylamide gel (ThermoFisher Scientific) at 150 V for 1.5 h at room temperature. Protein was transferred to an Immobilon PVDF membrane (ThermoFisher Scientific) in Tris-glycine transfer buffer (ThermoFisher Scientific) containing 10% methanol for ~ 18 h at room temperature and 0.03 A. Membranes were blocked in 2% milk in TBST overnight at 4 °C. The next day, membranes were transferred to primary antibody dilutions in 2% milk and incubated at 4 °C overnight. Primary antibodies included: anti-MPZ (rabbit; 1:5000; EMD Millipore, Burlington, MA), anti-cJun (rabbit; 1:5000; Cell Signaling Technology, Danvers, MA), anti-IARS (rabbit; 1:5000; GeneTex, Irvine, CA), anti-SOX10 (guinea pig; 1:2000; kind gift from Dr. Michael Wegner), anti-DDR1 (rabbit; 1:1000; Cell Signaling Technology, Danvers, MA), anti-actin (rabbit; 1:5000; Sigma-Aldrich, St. Louis, MO), anti-GAS7 (mouse; 1:100; Santa Cruz Biotechnology, Dallas, TX), and anti-AARS (rabbit; 1:2000; Bethyl Laboratories, Montgomery, TX). Secondary antibodies conjugated to horse radish peroxidase were diluted in 2% milk and incubated with membranes for one hour at room temperature. Antibodies included anti-rabbit HRP (donkey; 1:5000; EMD Millipore, Burlington, MA), anti-mouse HRP (goat; 1:2000; ThermoFisher Scientific), and anti-guinea pig HRP (goat; 1:5000; kind gift from Dr. Miriam Meisler). Membranes were incubated with West Dura HRP substrate (ThermoFisher Scientific) then exposed to X-ray film for between one second and three minutes. Protein band intensities were quantified with ImageJ [92]. The ratio of the band of interest to the protein loading control was calculated for each sample, then normalized to the control condition. The mean (bar height) and standard deviation (error bars) of normalized ratios are represented in the figures. Statistical comparisons were completed using the Student’s t-test (two-tailed).

### RNA isolation and RT-PCR

To probe gene expression profiles relevant for Schwann cells, we studied RNA from each model and condition. RNA was isolated from rat sciatic nerve, primary Schwann cells, and S16 cells using the RNeasy RNA isolation kit (Qiagen USA, Germantown, MD) according to the manufacturer’s protocol, with the addition of on-column RNase-free DNase treatment (Qiagen USA). RNA concentration was assessed using a Nanodrop Lite (ThermoFisher Scientific). For RT-PCR experiments, cDNA samples were generated using 1 μg of RNA and the High Capacity cDNA Reverse Transcription Kit (ThermoFisher Scientific) according to the manufacturer’s protocol. PCR was performed using PCR Supermix (ThermoFisher Scientific) or Phusion High-Fidelity PCR kit (New England Biolabs). Blank (cDNA-negative) controls were included for each primer pair and standard PCR conditions were used.

### Generation and analysis of Tn5Prime sequencing libraries

Tn5Prime was employed for the genome-wide identification and quantification of transcription start site use as a measure of promoter activity. Tn5Prime libraries were prepared as described by Cole and colleagues [26] starting with 5 ng total RNA and with the following exception. After the final PCR, samples were electrophoresed on a 0.8% low-melt agarose TAE gel. The gel was visualized on a Safe Imager Blue Light Transilluminator (ThermoFisher Scientific) and the 400 bp to 1 kb size region was excised. DNA was isolated using the Qiagen Gel Extraction Kit (Qiagen USA) according to manufacturer’s protocol. Sample yield was measured using the Qubit Broad Range kit for double-stranded DNA (ThermoFisher Scientific). Two Tn5Prime libraries were generated using RNA samples from two independent adult rat sciatic nerves (ages 6–9 months). Six libraries were generated using RNA isolated from independent populations of primary Schwann cells, three each that were CPT-cAMP- or control-treated. Two libraries were generated using two independent RNA samples from parental, unmodified S16 cells. Four libraries were generated using RNA samples from each of the four independent ΔSOX10 S16 clonal cell lines. Sequencing data from all samples were included in downstream analyses. Libraries were subjected to next-generation sequencing on Illumina HiSeq 4000 (Sciatic Nerve and S16 libraries) or NovaSeq (primary Schwann cell libraries) sequencers. Quality of data was assessed with FastQC (http://www.bioinformatics.babraham.ac.uk/projects/fastqc/). Adapter sequences were trimmed with Cutadapt [93], trimmed reads were mapped to the rat genome (rn5) with STAR [94], and BAM files were generated with SAMtools [95]. Reads were organized by start site using the Make_CTSS script from Takahashi and colleagues [96], and the resulting start site counts were clustered into defined transcription start sites (TSSs) using Paraclu [97]. Read counts per TSS per sample were generated using featureCounts [98] and statistical analysis was performed using edgeR [45]. Differentially expressed TSSs were defined by FDR-corrected *p*-values less than 0.05.

### Software and datasets employed for computational analyses

Several bioinformatic approaches were used to analyze the sequences surround SOX10-associated TSSs. Genomic coordinates for rat RefSeq (rn5) genes were extracted from the UCSC Genome Browser [99]. TSSs were assigned to genes using BEDTools [100], requiring the TSS to map within 1 kilobase and on the same strand as the gene. To account for poor gene annotation in the rat genome, TSSs that did not map to a rat gene were converted to the orthologous mouse coordinates (mm10) using the liftOver executable from the UCSC Genome Browser [99] and mapped to mouse RefSeq genes in the same manner. H3K4me3 ChIP-Seq peaks from rat scatic nerve [28], SOX10 ChIP-Seq peaks from rat sciatic nerve [19], and TSSs defined by Tn5Prime in sciatic nerve (see above) were intersected using BEDTools to define TSSs mapping within 1 kilobase of H3K4me3 and/or SOX10 ChIP-Seq peaks. Human RefSeq transcript annotations (hg38) for loci associated with SOX10-bound promoters were downloaded through the UCSC Genome Browser [99] and manually curated for transcription start site and coding sequence diversity. Empirical cumulative distribution function curves for TSS expression levels were generated using the plot() and ecdf() functions in R [101]. Statistical analysis was performed using the Mann-Whitney U-test. Aggregate analysis of SOX10 ChIP-Seq data surrounding TSSs was performed using metagene [30] with permutation-based statistical analysis using similaRpeak (https://github.com/adeschen/similaRpeak) based on the area under the curve. Gene ontology analyses were performed using geneontology.org [102, 103]. Enriched terms were defined by corrected p-value < 0.05. Heatmaps organized by gene ontology terms were generated using Heatmapper [104]. Expression values were Z-score scaled per TSS. Genomic sequences of regions surrounding TSSs were extracted using the UCSC Genome Browser [99]. SOX10, TATA Box, and Initiator motifs were identified using custom pearl scripts (github.com/efogarty/Schwann-Cell-SOX10-Promoters) in Bioperl [105]. Conservation scores for SOX10 motif sequences were extracted from the UCSC Genome Browser rn5 13-way PhastCons data file [99] using a publicly available custom script written by Dr. Ian Donaldson, University of Manchester (https://www.biostars.org/p/16724/#16731). Per-base PhastCons scores were averaged to assign per-motif scores. Box plots were generated using BoxPlotR [106]. GC content and CpG islands were defined by the EMBOSS freak and cpgplot tools, respectively [107]. CpG islands were defined using cpgplot default parameters. CAGE data from 11 mouse tissues including cortex, spinal cord, skin, lung, heart, colon, thymus, stomach, liver, ovary, and testis were downloaded from FANTOM5 [49]. TSS genomic coordinates were converted to the orthologous mouse (mm10) coordinates using liftOver from the UCSC Genome Browser [99] and featureCounts was used to quantify read counts in each tissue. TSS-specific Tau scores were calculated as described by Yanai and colleagues [50]. Statistical analyses of distributions of SOX10 motifs, motif conservation, and Tau scores were performed using the Mann-Whitney U-test.

### Luciferase reporter constructs and promoter activity assays

To test genomic elements for regulatory activity, we employed luciferase reporter assays. Oligonucleotide primers containing attB1 and attB2 Gateway cloning sequences (Invitrogen Life Technologies/ThermoFisher Scientific) were designed for PCR-based amplification of promoter regions of interest. The regions were amplified from human genomic DNA using PCR Supermix (ThermoFisher Scientific) or Phusion High-Fidelity Polymerase (New England Biotechnology). Subsequent to PCR amplification and purification, each genomic segment was cloned into the pDONR221 vector using BP Clonase (Invitrogen). Resulting constructs were genotyped by digestion with BsrGI (New England Biolabs, Ipswich, MA) and subjected to DNA sequence analysis to ensure the integrity of the insert. The resulting pDONR221 construct was recombined with an expression construct (pE1B-luciferase) [108] using LR Clonase (Invitrogen) to clone each region upstream of a minimal promoter directing expression of a luciferase reporter gene. Successful recombination was confirmed via digestion of DNA with BsrG1. Site-directed mutagenesis was performed using the QuikChange II XL Site-Directed Mutagenesis Kit (Agilent Technologies, Inc., Santa Clara, CA). Mutagenesis primers were designed to delete the SOX10 binding site(s) within each element. Mutagenesis was performed in the pDONR221 construct and DNA from each resulting clone underwent sequence analysis to verify that only the desired mutation was produced. Verified clones were recombined into pE1B-luciferase using LR Clonase (Invitrogen).

Unmodified parental S16 cells [36] were grown under standard conditions in DMEM plus 10% fetal bovine serum, 2 mM L-glutamine, 50 U/mL penicillin, and 50 g/mL streptomycin. For luciferase assays, ~ 1000 cells were plated in each well of a 96-well plate. Cells were cultured for 24 h under standard conditions prior to transfections. Lipofectamine 2000 (ThermoFisher Scientific) was diluted 1:100 in OptiMEM I reduced serum medium (ThermoFisher Scientific) and incubated at room temperature for 10 min. Each DNA construct to be transfected was individually diluted in OptiMEM to a concentration of 8 ng/μL. An internal control renilla construct was added to the solution at 8 pg/μL. One volume of lipofectamine solution was added to each DNA solution and allowed to sit for 20 min at room temperature. Cells were incubated with transfection solution for 4 h under standard conditions and then the medium was changed to standard growth medium. 48 h after transfection cells were washed with 1X PBS and lysed for 1 h shaking at room temperature using 20 μL 1X Passive Lysis Buffer (Promega, Madison, WI). 10 μL of lysate from each well was transferred into a white polystyrene 96-well plate (Corning Inc., Corning, NY). Luciferase and renilla activities were determined using the Dual Luciferase Reporter 1000 Assay System (Promega) and a Glomax Multi-Detection System (Promega). Each reaction was performed at least 24 times. The ratio of luciferase to renilla activity and the fold change in this ratio compared to a control luciferase expression vector with no genomic insert were calculated. The mean (bar height) and standard deviation (error bars) of the fold difference are represented in the figures. Statistical comparisons were completed using the Student’s t-test (two-tailed).

## Supplementary information

**Additional file 1: Supplementary Figure 1.** Validation of CPT-cAMP-induced differentiation of primary Schwann cells. Protein lysates from independent populations of primary Schwann cells treated with CPT-cAMP (cAMP) or vehicle (Control). MPZ was used a positive marker of differentiation, while cJun serves as a negative marker of differentiation. IARS was used as a protein loading control. Numbered dashes between blots indicate the position of protein size markers in kilodaltons (kDa). **Supplementary Figure 2.** Generation of ΔSOX10 S16 cell model. (A) Locations of guide RNAs designed against the first coding exon of the rat *Sox10* locus. (B) RT-PCR to assay *Sox10* transcript expression in unmodified, parental S16 cells and each individual ΔSOX10 S16 clone. Two independent primer sets for *Sox10* and primers for *Actb* as a positive control. Blank reactions (no cDNA) were included for each primer pair. Sizes of DNA ladder are shown to the left in base pairs (bp). (C) SOX10 protein expression in unmodified, parental S16 cells and each individual ΔSOX10 S16 clone. IARS was used as a protein loading control. Numbered dashes indicate positions of protein size markers in kilodaltons (kDa). **Supplementary Figure 3.** SOX10-dependent transcription start sites show no difference in GC content. (A). GC content (y-axis) averaged in 10 base pair bins for genomic regions surrounding transcription start sites (TSSs) that were downregulated, upregulated, or unchanged in ΔSOX10 S16 cells (Fig. 3a). X-axis: distance from the TSS (bp, base pairs). (B) The fraction of downregulated, upregulated, and unchanged TSSs (Fig. 3a) that fall into quintile bins based on GC content measured across the +/− 1 kb window surrounding the TSS. **Supplementary Figure 4.** SOX10-dependent transcription start sites are associated with increased SOX10 ChIP-Seq signal independent of GC content. Aggregate SOX10 ChIP-Seq data in the 2-kilobase region surrounding TSSs that were downregulated, upregulated, or unchanged in ΔSOX10 S16 cells as in Fig. 3a, binned by GC content as in Supplementary Figure 3B. X-axis: genomic distance from the TSS (base pairs, bp). Y-axis: average SOX10 ChIP-Seq signal (RPM, reads per million). Asterisk indicates *p* < 0.05. **Supplementary Figure 5.** SOX10-dependent TSSs exhibit highest expression in tissues containing SOX10-positive cells. Y-axis: distributions of per-TSS normalized expression profile component in each tissue. The normalized expression profile component score is 1 for the tissue where the TSS is most highly expressed. Expression values in other tissues are normalized to this value. Whiskers extend to the 5th and 95th percentile of the data. **Supplementary Figure 6.***ARPC1A* transcript sequences. (A) The human *ARPC1A* locus is annotated with two RefSeq transcript isoforms, both originating at exon 1A (‘1A’ in panel). Red box indicates the location of the Tn5Prime-defined TSS as in Fig. 5a. (B) The rat *Arpc1a* locus is shown with exons 7, 8, and 10 indicated. The locations of RT-PCR primers used in panel C are shown by vertical black bars. The sequence gap in the rat genome which omits exon 9 is shown by the thick black horizontal bar. (C) RT-PCR was used to validate the expression of a spliced *Arpc1a* transcript with the expected architecture using cDNA from rat sciatic nerve. A blank reaction (no cDNA) was used as a negative control. Sizes of DNA ladder markers are indicated to the left in base pairs (bp). (D) The mouse *Arpc1a* locus is shown with exons 7, 8, 9, and 10 indicated. The rat sciatic nerve-derived transcript sequence mapped to the mouse genome as shown below. **Supplementary Figure 7.***CHN2* transcript sequences. (A) The human *CHN2* locus is annotated with five RefSeq transcript start sites, originating at exons 1A through 1E (‘1A’ through ‘1E’ in panel). Red box indicates the location of the Tn5Prime-defined TSS as in Fig. 6a. (B) The rat *Chn2* locus is shown, with exon 1D indicated. The locations of RT-PCR primers used in panel C are shown by vertical black bars. The rat sciatic nerve-derived transcript sequence mapped to the rat genome as shown at the bottom of the panel. (C) RT-PCR was used to validate the expression of a spliced *Chn2* transcript with the expected architecture using cDNA from rat sciatic nerve. A blank reaction (no cDNA) was used as a negative control. Sizes of DNA ladder markers are indicated to the left in base pairs (bp). **Supplementary Figure 8.***DDR1* transcript sequences. (A) The human *DDR1* locus is annotated with five RefSeq transcript start sites, originating at exons 1A through 2 (‘1A’ through ‘2’ in panel). Red box indicates the location of the Tn5Prime-defined TSS as in Fig. 7a. (B) The rat *Ddr1* locus is shown. The locations of RT-PCR primers used in panel C are shown by vertical black bars. The rat sciatic nerve-derived transcript sequences mapped to the rat genome as shown at the bottom of the panel. (C) RT-PCR was used to validate the expression of a spliced *Ddr1* transcript with the expected architecture using cDNA from rat sciatic nerve. A blank reaction (no cDNA) was used as a negative control. Sizes of DNA ladder markers are indicated to the left in base pairs (bp). **Supplementary Figure 9.***GAS7* transcript sequences. (A) The human *GAS7* locus is annotated with four RefSeq transcript start sites, originating at exons 1A through 1D (‘1A’ through ‘1D’ in panel). Red box indicates the location of the Tn5Prime-defined TSS as in Fig. 8a. (B) The rat *Gas7* locus is shown with the locations of RT-PCR primers used in panel C indicated by vertical black bars. The rat sciatic nerve-derived transcript sequence mapped to the rat genome as shown at the bottom of the panel. (C) RT-PCR was used to validate the expression of exon 1B-derived *Gas7* transcripts with the expected architecture using cDNA from rat sciatic nerve. A blank reaction (no cDNA) was used as a negative control. Sizes of DNA ladder markers are indicated to the left in base pairs (bp). **Supplementary Figure 10.***Gas7* exon 1B is the predominant transcription start site utilized in Schwann cells. The rat *Gas7* genomic locus. Y-axes for H3K4me3 and SOX10 ChIP-Seq data: fold enrichment of sequencing reads above chromatin input. Below, the locations of each first exon from the human *GAS7* locus (see Supplementary Figure 8A), converted to rat genomic coordinates. Y-axes for Tn5Prime data from rat sciatic nerve, CPT-cAMP- (cAMP) and vehicle-treated (Control) primary Schwann cells, and unmodified and ΔSOX10 S16 cells: number of transcript 5’ends mapped per base, in reads per million.

**Additional file 2: Supplementary Table 1.** Coordinates and supporting information for SOX10-associated transcription start sites (TSSs) in each model. First tab: All 4993 TSSs associated with SOX10 and H3K4me3 ChIP-Seq peaks in sciatic nerve. Second tab: 465 TSSs that increased in expression upon cAMP treatment in primary Schwann cells. Third tab: 401 TSSs that decreased in expression upon cAMP treatment in primary Schwann cells. Fourth tab: 265 TSSs that decreased in expression upon loss of SOX10 in S16 cells. Fifth tab: 100 TSSs that increased in expression upon loss of SOX10 in S16 cells.

**Additional file 3: Supplementary Table 2.** Genes that harbor SOX10-associated transcription start sites (TSSs) and are annotated with multiple isoforms in the human RefSeq database. First column: A list of all genes that harbor a SOX10-associated TSS (reside near SOX10 and H3K4me3 ChIP-Seq peaks) and are annotated with at least two unique transcript isoforms in human RefSeq. Second column: A list of all genes that harbor a SOX10-associated TSS and are annotated with at least two unique transcript isoforms in human RefSeq that also confer unique protein-coding sequences. Third column: A list of all genes that harbor a SOX10-dependent TSS (exhibited reduced expression upon loss of SOX10 in S16 cells) and are annotated with at least two unique transcript isoforms in human RefSeq. Fourth column: A list of all genes that harbor a SOX10-dependent TSS and are annotated with at least two unique transcript isoforms in human RefSeq that also confer unique protein-coding sequences.

**Additional file 4: Supplementary Table 3.** Comparison of genes harboring SOX10-dependent TSSs in S16 cells in the current study to findings from Srinivasan et al., 2012. The 169 genes that were found to harbor a TSS that was SOX10-dependent in the S16 cell model (Tn5Prime-based analysis, using TSSs that were expressed in sciatic nerve and reside near SOX10 and H3K4me3 ChIP-Seq peaks) were compared to genes that were downregulated by SOX10 siRNA in S16 cells by Srinivasan et al. (microarray-based analysis).

**Additional file 5: Supplementary Table 4.** Genomic elements tested for SOX10-dependent regulatory activity in luciferase assays.

## Data Availability

The Tn5Prime data discussed in this publication have been deposited in NCBI’s Gene Expression Omnibus and are accessible through GEO Series accession number GSE139321. Other data and materials are available from the corresponding author upon request. Previously published datasets utilized in these analyses include SOX10 ChIP-Seq from rat sciatic nerve (GSE64703), H3K4me3 ChIP-Seq from rat sciatic nerve (GSE84272), and CAGE data from mouse tissues (https://fantom.gsc.riken.jp/5/datafiles/latest/basic/mouse.tissue.hCAGE/).

## Abbreviations

TSS: Transcription start site
ChIP-Seq: Chromatin Immunoprecipitation with next-generation sequencing
PNS: Peripheral nervous system
CAGE: Cap analysis of gene expression
CPT-cAMP: (4-Chlorophenylthio) adenosine 3′,5′-cyclic monophosphate
