# Supplementary material for: SOX10-regulated promoter use defines isoform-specific gene expression in Schwann cells
Source: BMC Genomics. 2020 Aug 8;21:549. doi: 10.1186/s12864-020-06963-7 (PMC7430845; doi:10.1186/s12864-020-06963-7)
Supplement: Supplementary file 1 — Additional file 1: Supplementary Figure 1. Validation of CPT-cAMP-induced differentiation of primary Schwann cells. Protein lysates from independent populations of primary Schwann cells treated with CPT-cAMP (cAMP) or vehicle (Control). MPZ was used a positive marker of differentiation, while cJun serves as a negative marker of differentiation. IARS was used as a protein loading control. Numbered dashes between blots indicate the position of protein size markers in kilodaltons (kDa). Supplementary Figure 2. Generation of ΔSOX10 S16 cell model. (A) Locations of guide RNAs designed against the first coding exon of the rat Sox10 locus. (B) RT-PCR to assay Sox10 transcript expression in unmodified, parental S16 cells and each individual ΔSOX10 S16 clone. Two independent primer sets for Sox10 and primers for Actb as a positive control. Blank reactions (no cDNA) were included for each primer pair. Sizes of DNA ladder are shown to the left in base pairs (bp). (C) SOX10 protein expression in unmodified, parental S16 cells and each individual ΔSOX10 S16 clone. IARS was used as a protein loading control. Numbered dashes indicate positions of protein size markers in kilodaltons (kDa). Supplementary Figure 3. SOX10-dependent transcription start sites show no difference in GC content. (A). GC content (y-axis) averaged in 10 base pair bins for genomic regions surrounding transcription start sites (TSSs) that were downregulated, upregulated, or unchanged in ΔSOX10 S16 cells (Fig. 3a). X-axis: distance from the TSS (bp, base pairs). (B) The fraction of downregulated, upregulated, and unchanged TSSs (Fig. 3a) that fall into quintile bins based on GC content measured across the +/− 1 kb window surrounding the TSS. Supplementary Figure 4. SOX10-dependent transcription start sites are associated with increased SOX10 ChIP-Seq signal independent of GC content. Aggregate SOX10 ChIP-Seq data in the 2-kilobase region surrounding TSSs that were downregulated, upregulated, or unchanged in ΔSO [file 12864_2020_6963_MOESM1_ESM.docx]

**Supplementary Figure 1**

**
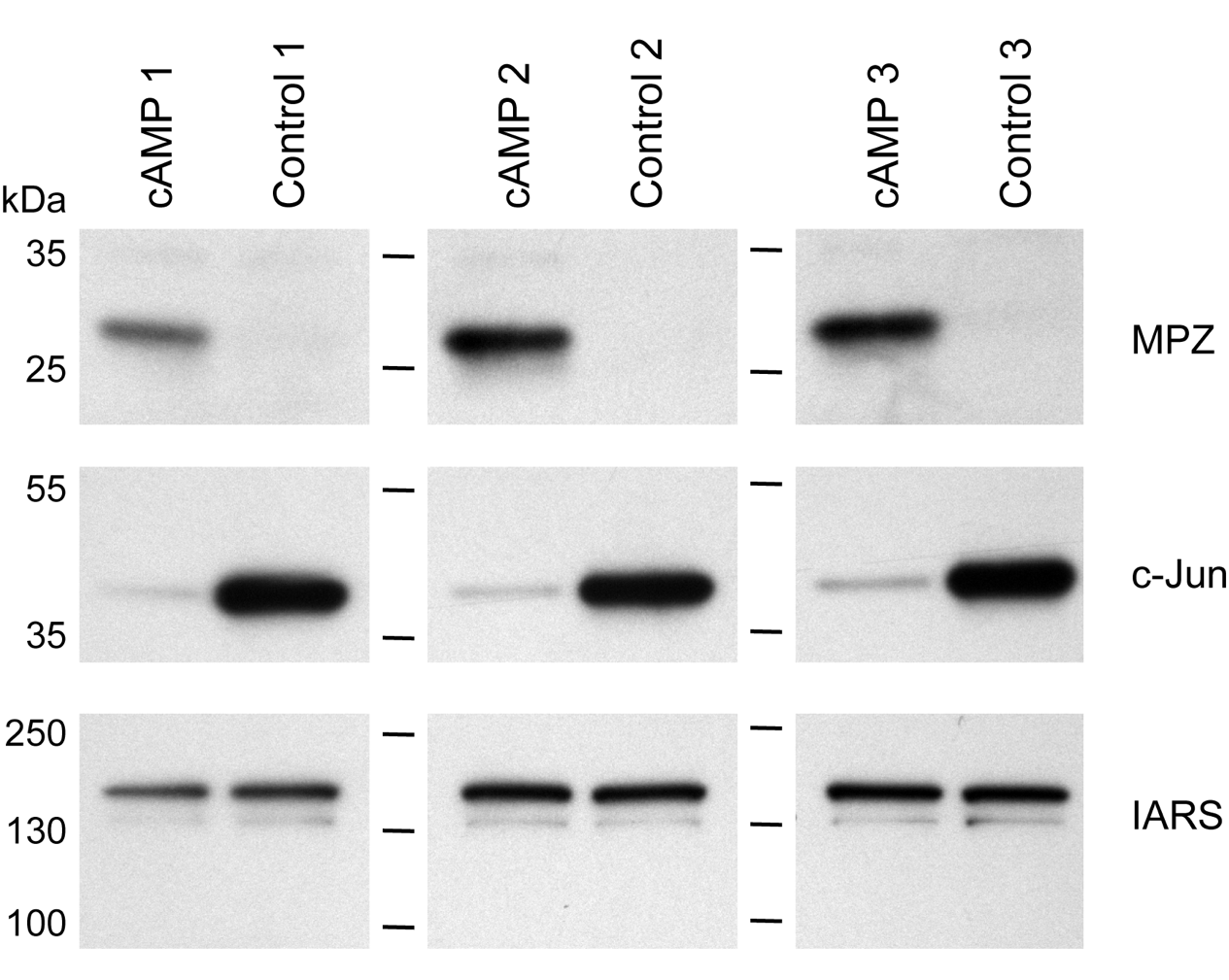
**

Validation of CPT-cAMP-induced differentiation of primary Schwann cells.

Protein lysates from independent populations of primary Schwann cells treated with CPT-cAMP (cAMP) or vehicle (Control). MPZ was used a positive marker of differentiation, while cJun serves as a negative marker of differentiation. IARS was used as a protein loading control. Numbered dashes between blots indicate the position of protein size markers in kilodaltons (kDa).

**Supplementary Figure 2**

**
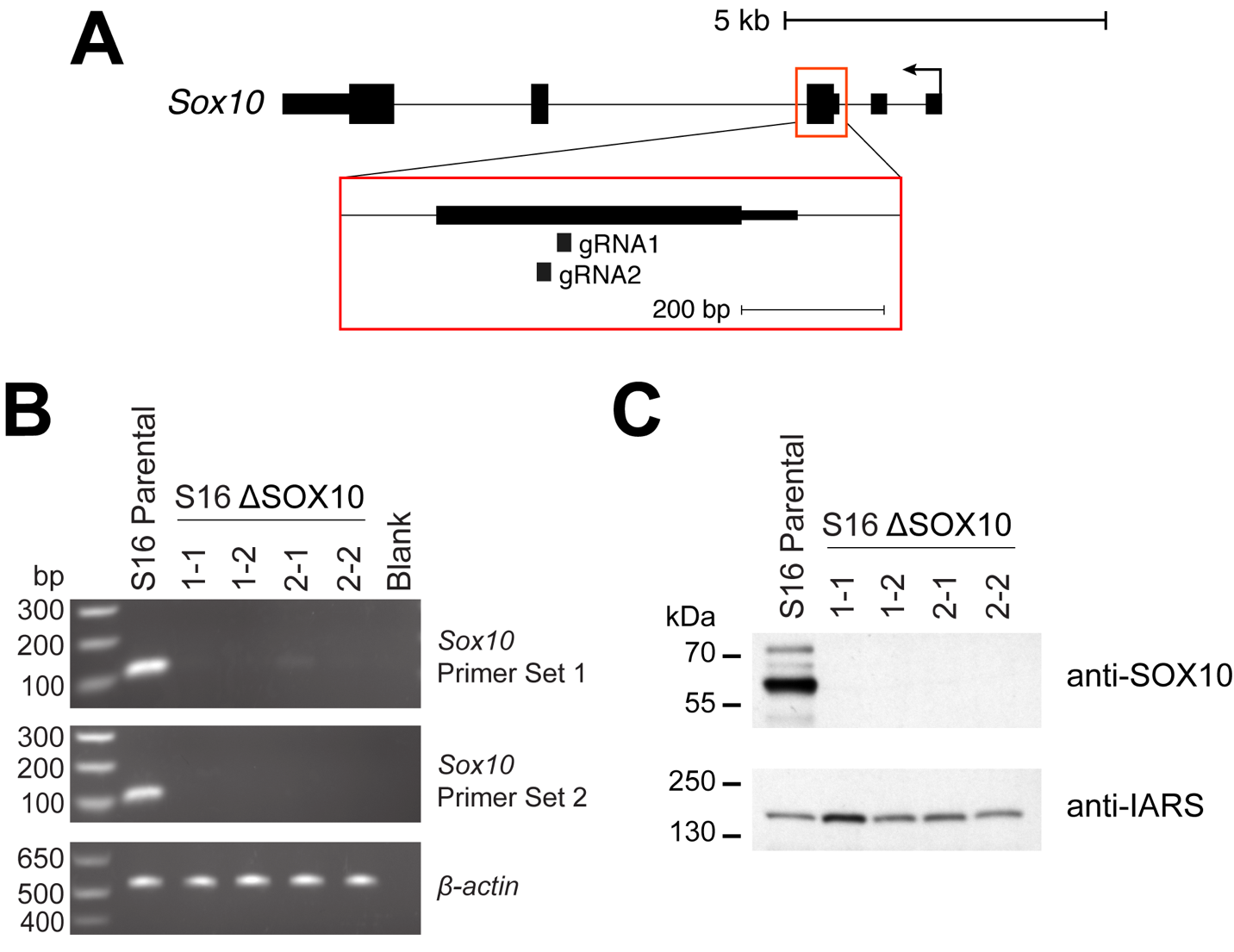
**

Generation of ΔSOX10 S16 cell model.

(**A**) Locations of guide RNAs designed against the first coding exon of the rat *Sox10* locus. (**B**) RT-PCR to assay *Sox10* transcript expression in unmodified, parental S16 cells and each individual ΔSOX10 S16 clone. Two independent primer sets for *Sox10* and primers for *Actb* as a positive control. Blank reactions (no cDNA) were included for each primer pair. Sizes of DNA ladder are shown to the left in base pairs (bp). (**C**) SOX10 protein expression in unmodified, parental S16 cells and each individual ΔSOX10 S16 clone. IARS was used as a protein loading control. Numbered dashes indicate positions of protein size markers in kilodaltons (kDa).

**Supplementary Figure 3**

**
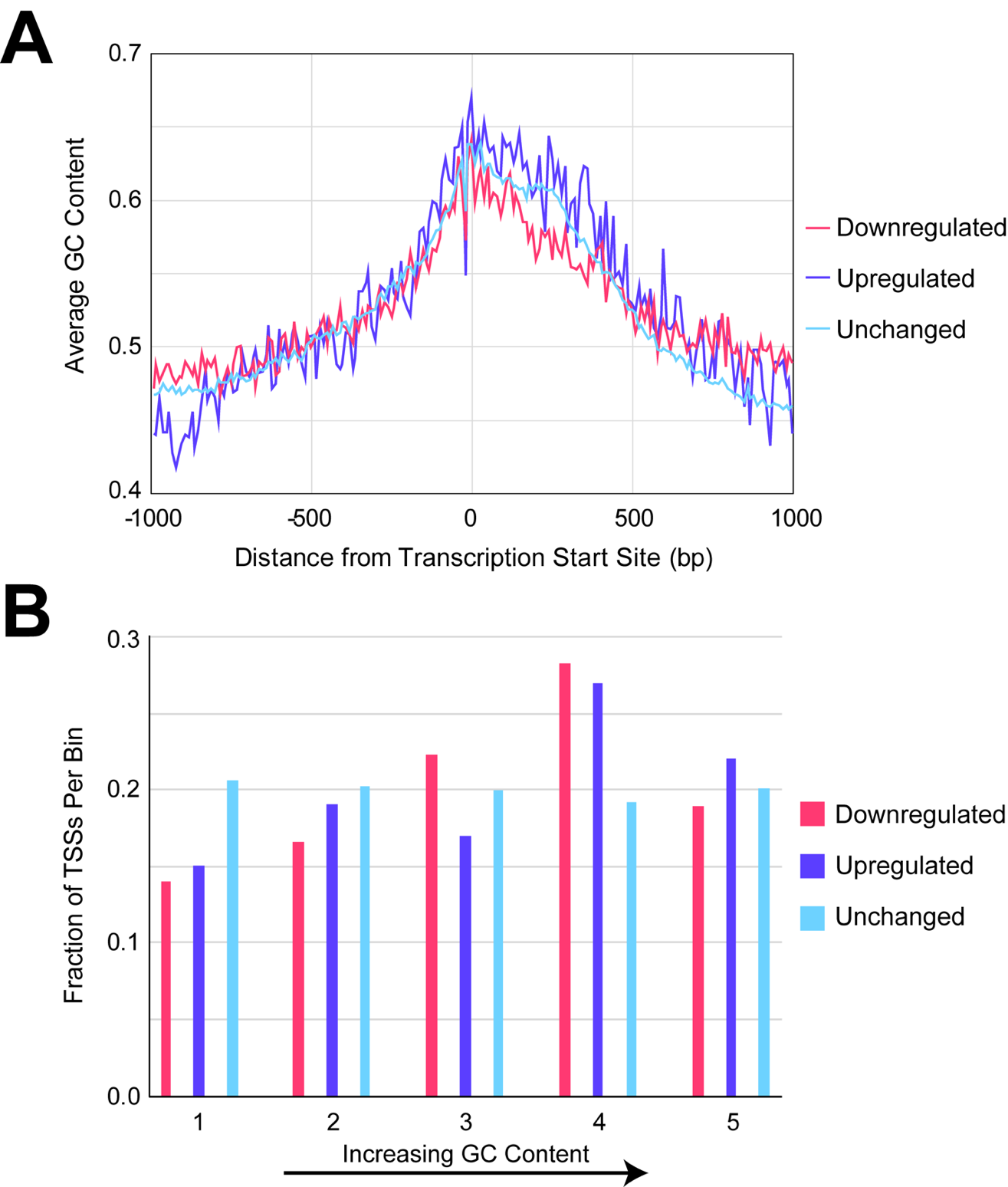
**

SOX10-dependent transcription start sites show no difference in GC content. (**A**)

GC content (y-axis) averaged in 10 base pair bins for genomic regions surrounding transcription start sites (TSSs) that were downregulated, upregulated, or unchanged in ΔSOX10 S16 cells (Figure 3A). X-axis: distance from the TSS (bp, base pairs). (**B**) The fraction of downregulated, upregulated, and unchanged TSSs (Figure 3A) that fall into quintile bins based on GC content measured across the +/- 1 kb window surrounding the TSS.

**Supplementary Figure 4**

**
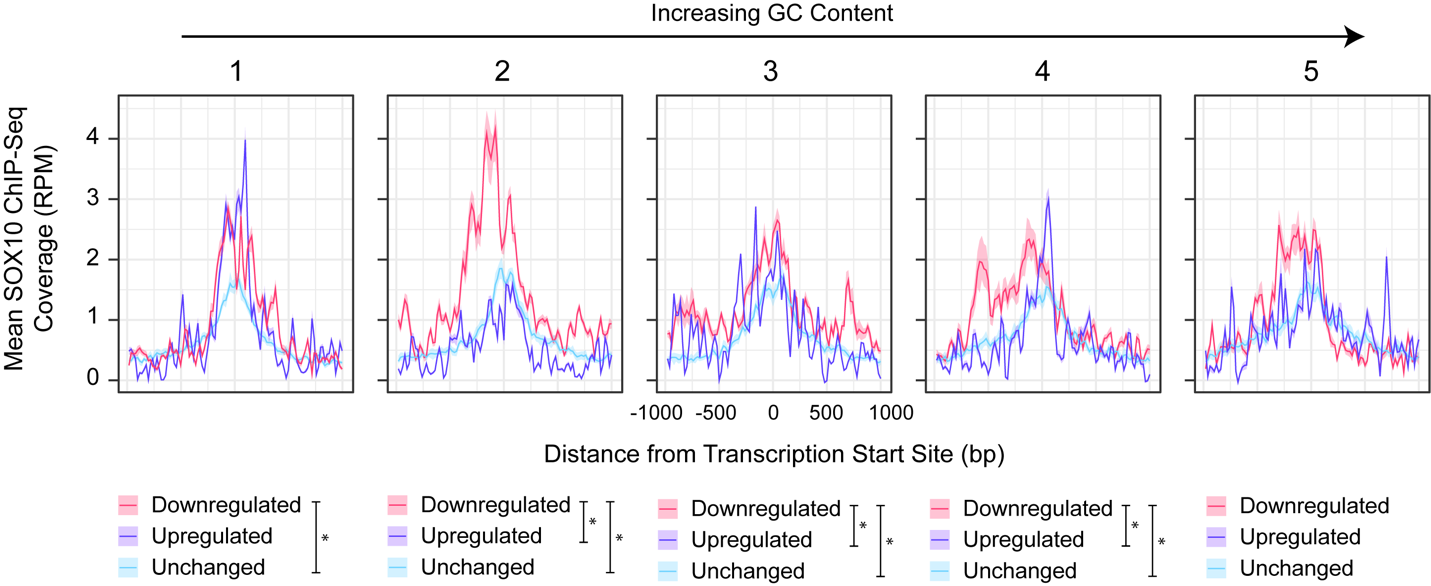
**

SOX10-dependent transcription start sites are associated with increased SOX10 ChIP-Seq signal independent of GC content.

Aggregate SOX10 ChIP-Seq data in the 2-kilobase region surrounding TSSs that were downregulated, upregulated, or unchanged in ΔSOX10 S16 cells as in Figure 3A, binned by GC content as in Supplementary Figure 3B. X-axis: genomic distance from the TSS (base pairs, bp). Y-axis: average SOX10 ChIP-Seq signal (RPM, reads per million). Asterisk indicates p < 0.05.

**Supplementary Figure 5**

**
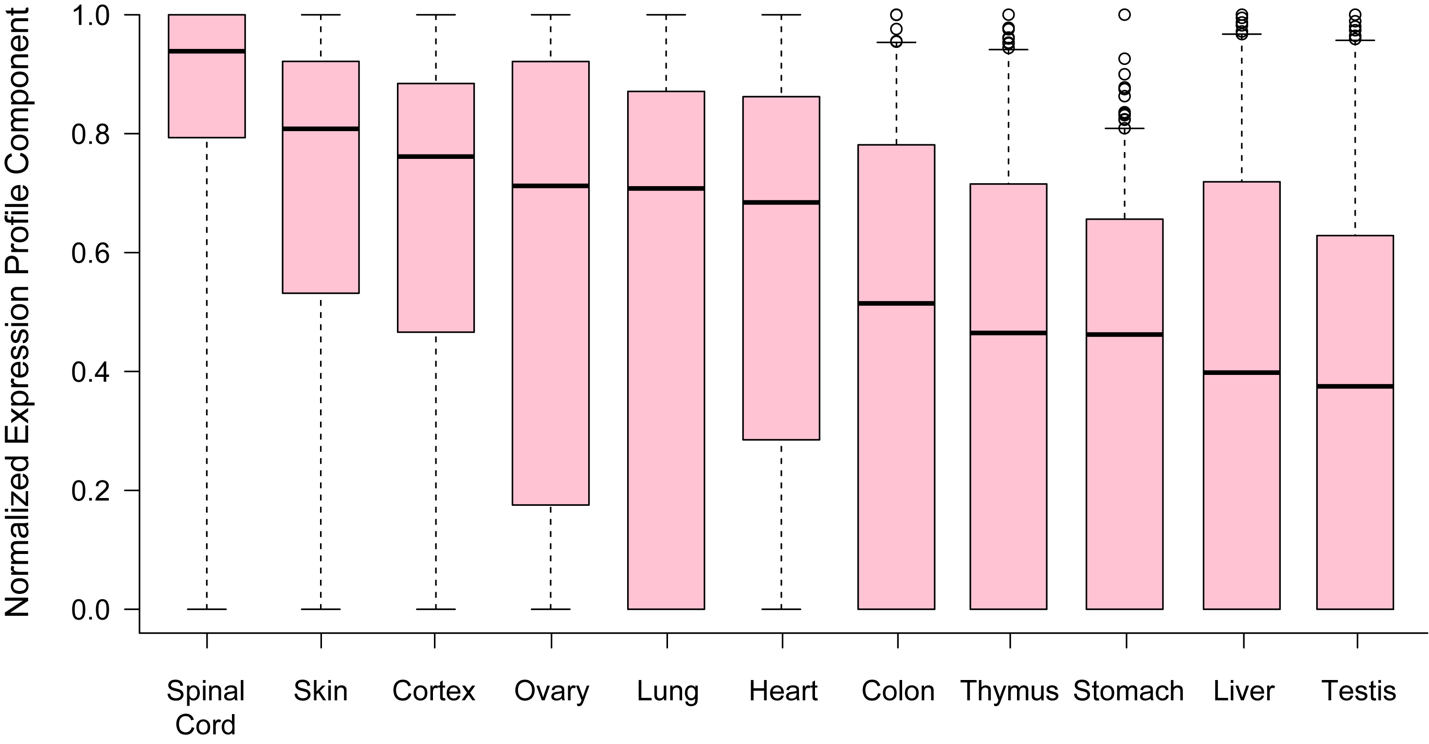
**

SOX10-dependent TSSs exhibit highest expression in tissues containing SOX10-positive cells. Y-axis: distributions of per-TSS normalized expression profile component in each tissue. The normalized expression profile component score is 1 for the tissue where the TSS is most highly expressed. Expression values in other tissues are normalized to this value. Whiskers extend to the 5^th^ and 95^th^ percentile of the data.

**Supplementary Figure 6**

**
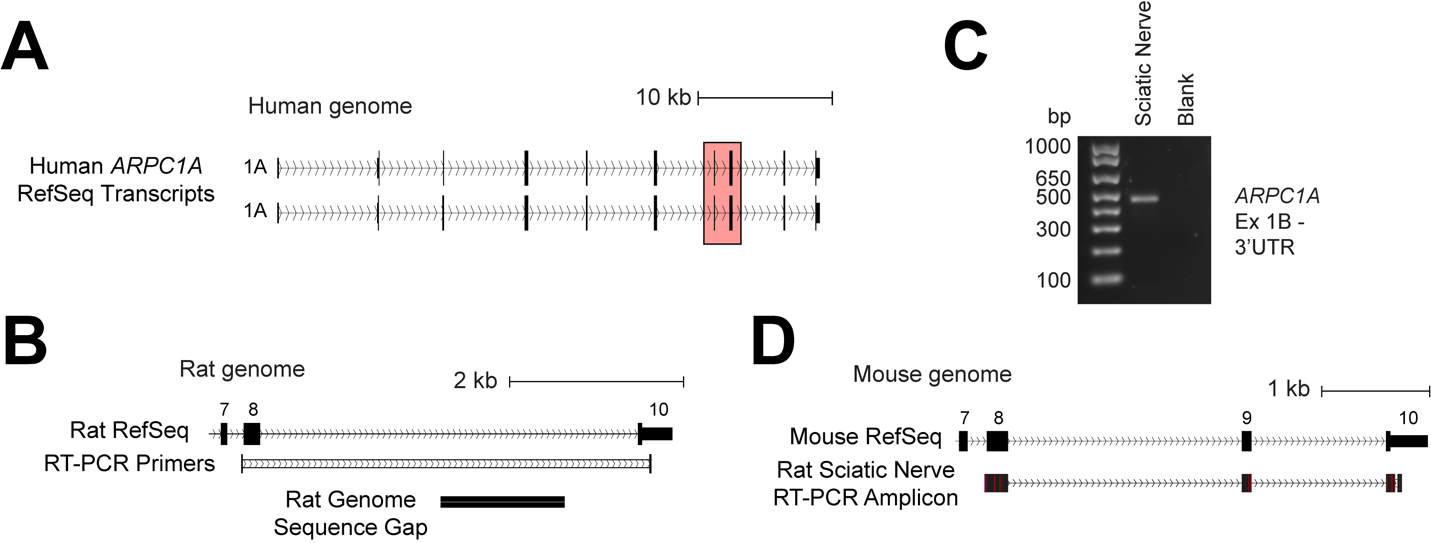
**

*ARPC1A* transcript sequences. (**A**) The human *ARPC1A* locus is annotated with two RefSeq transcript isoforms, both originating at exon 1A (‘1A’ in panel). Red box indicates the location of the Tn5Prime-defined TSS as in Figure 5A. (**B**) The rat *Arpc1a* locus is shown with exons 7, 8, and 10 indicated. The locations of RT-PCR primers used in panel C are shown by vertical black bars. The sequence gap in the rat genome which omits exon 9 is shown by the thick black horizontal bar. (**C**) RT-PCR was used to validate the expression of a spliced *Arpc1a* transcript with the expected architecture using cDNA from rat sciatic nerve. A blank reaction (no cDNA) was used as a negative control. Sizes of DNA ladder markers are indicated to the left in base pairs (bp). (**D**) The mouse *Arpc1a* locus is shown with exons 7, 8, 9, and 10 indicated. The rat sciatic nerve-derived transcript sequence mapped to the mouse genome as shown below.

**Supplementary Figure 7**

**
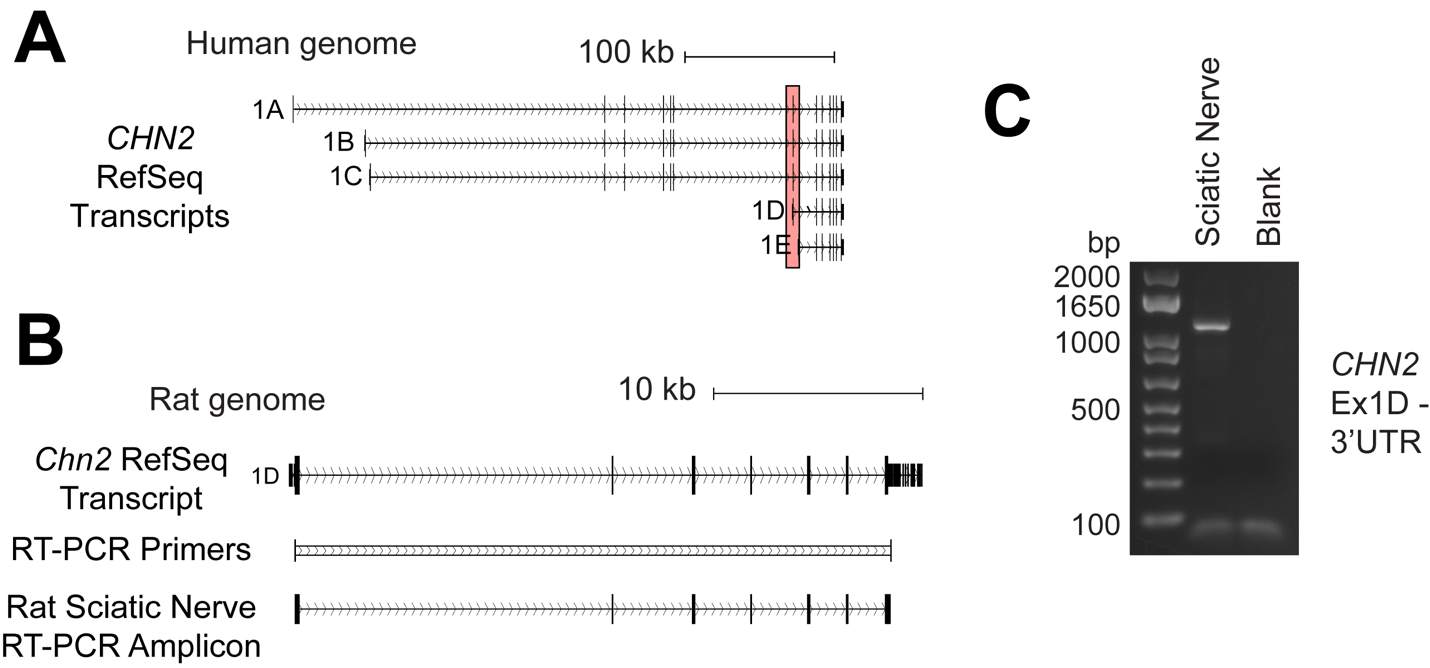
**

*CHN2* transcript sequences. (**A**) The human *CHN2* locus is annotated with five RefSeq transcript start sites, originating at exons 1A through 1E (‘1A’ through ‘1E’ in panel). Red box indicates the location of the Tn5Prime-defined TSS as in Figure 6A. (**B**) The rat *Chn2* locus is shown, with exon 1D indicated. The locations of RT-PCR primers used in panel C are shown by vertical black bars. The rat sciatic nerve-derived transcript sequence mapped to the rat genome as shown at the bottom of the panel. (**C**) RT-PCR was used to validate the expression of a spliced *Chn2* transcript with the expected architecture using cDNA from rat sciatic nerve. A blank reaction (no cDNA) was used as a negative control. Sizes of DNA ladder markers are indicated to the left in base pairs (bp).

**Supplementary Figure 8**

**
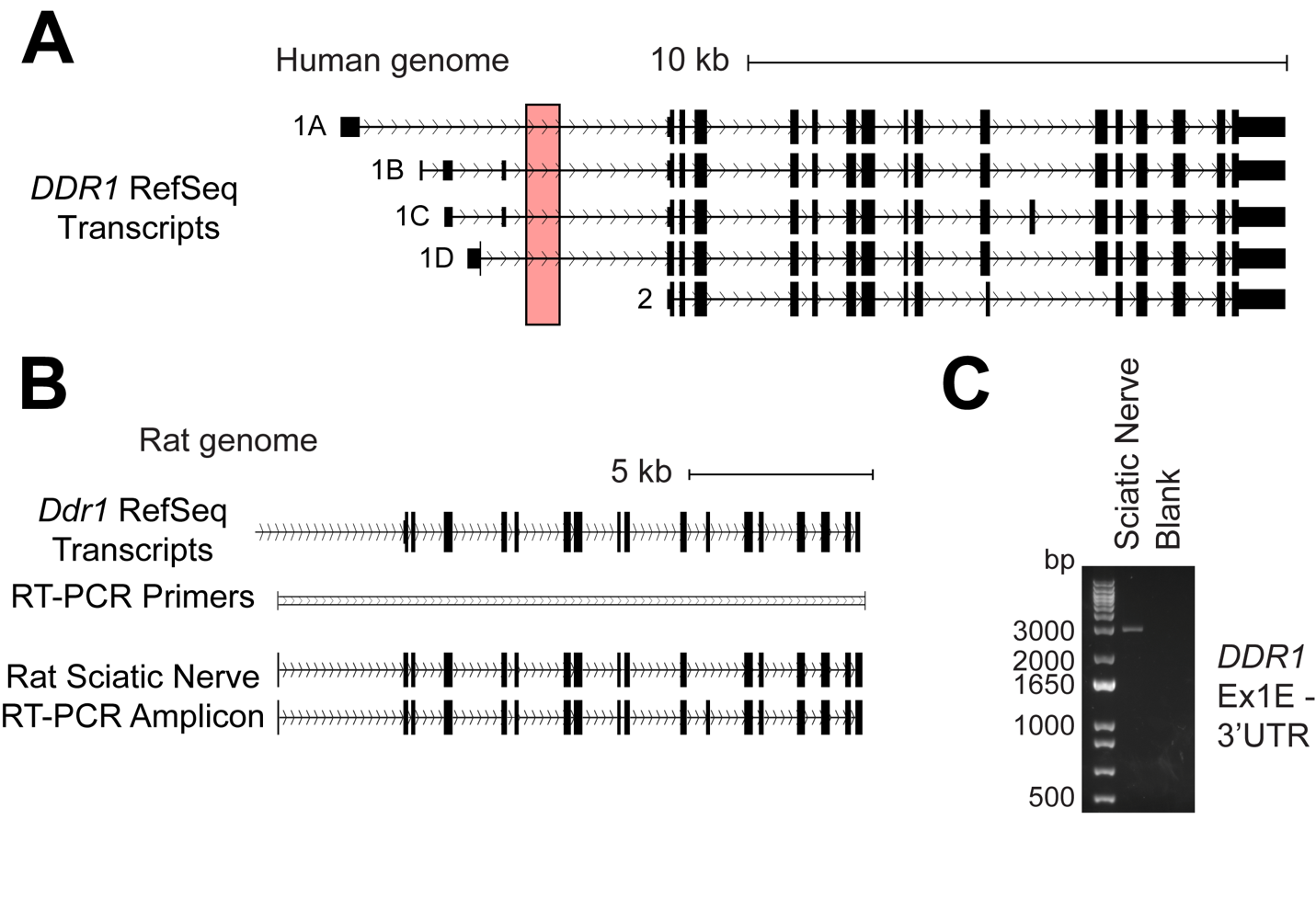
**

*DDR1* transcript sequences. (**A**) The human *DDR1* locus is annotated with five RefSeq transcript start sites, originating at exons 1A through 2 (‘1A’ through ‘2’ in panel). Red box indicates the location of the Tn5Prime-defined TSS as in Figure 7A. (**B**) The rat *Ddr1* locus is shown. The locations of RT-PCR primers used in panel C are shown by vertical black bars. The rat sciatic nerve-derived transcript sequences mapped to the rat genome as shown at the bottom of the panel. (**C**) RT-PCR was used to validate the expression of a spliced *Ddr1* transcript with the expected architecture using cDNA from rat sciatic nerve. A blank reaction (no cDNA) was used as a negative control. Sizes of DNA ladder markers are indicated to the left in base pairs (bp).

**Supplementary Figure 9**

**
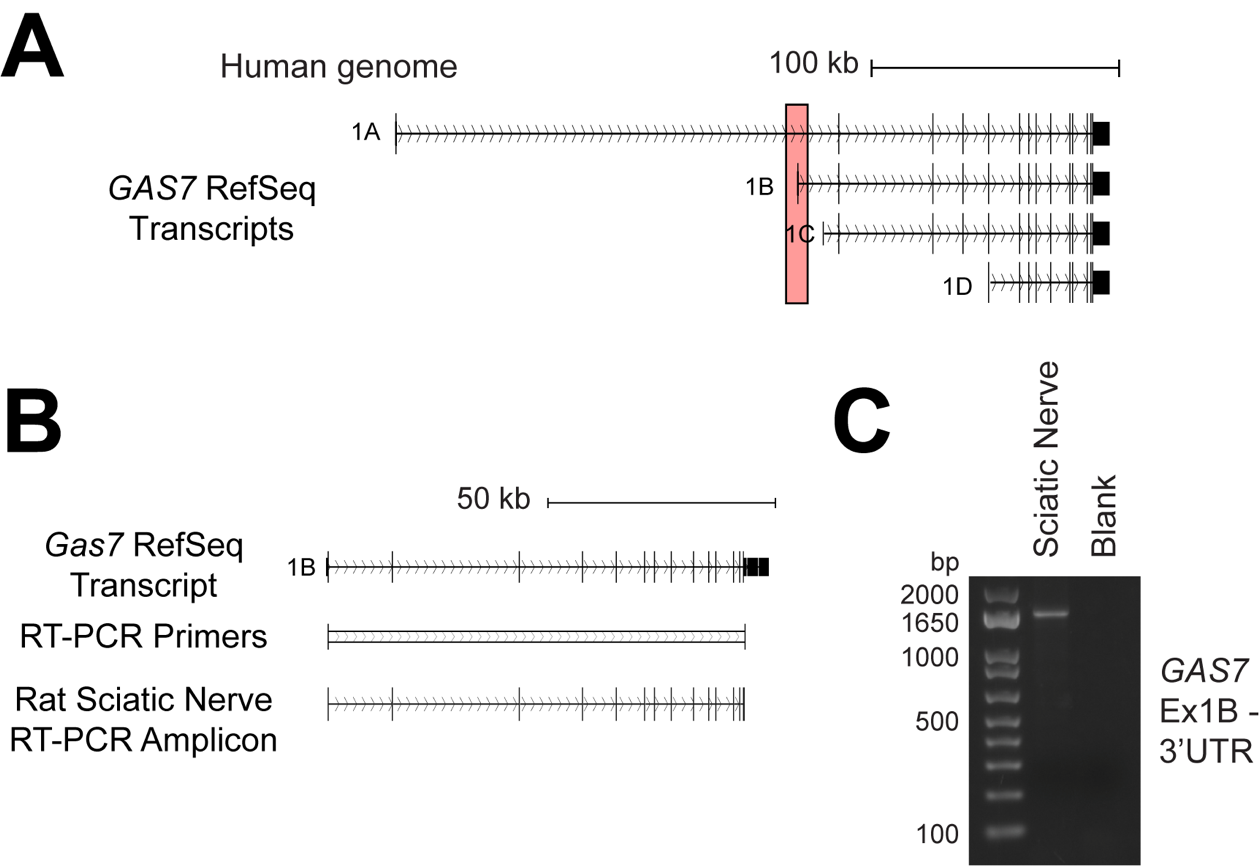
**

*GAS7* transcript sequences. (**A**) The human *GAS7* locus is annotated with four RefSeq transcript start sites, originating at exons 1A through 1D (‘1A’ through ‘1D’ in panel). Red box indicates the location of the Tn5Prime-defined TSS as in Figure 8A. (**B**) The rat *Gas7* locus is shown with the locations of RT-PCR primers used in panel C indicated by vertical black bars. The rat sciatic nerve-derived transcript sequence mapped to the rat genome as shown at the bottom of the panel. (**C**) RT-PCR was used to validate the expression of exon 1B-derived *Gas7* transcripts with the expected architecture using cDNA from rat sciatic nerve. A blank reaction (no cDNA) was used as a negative control. Sizes of DNA ladder markers are indicated to the left in base pairs (bp).

**Supplementary Figure 10**

**
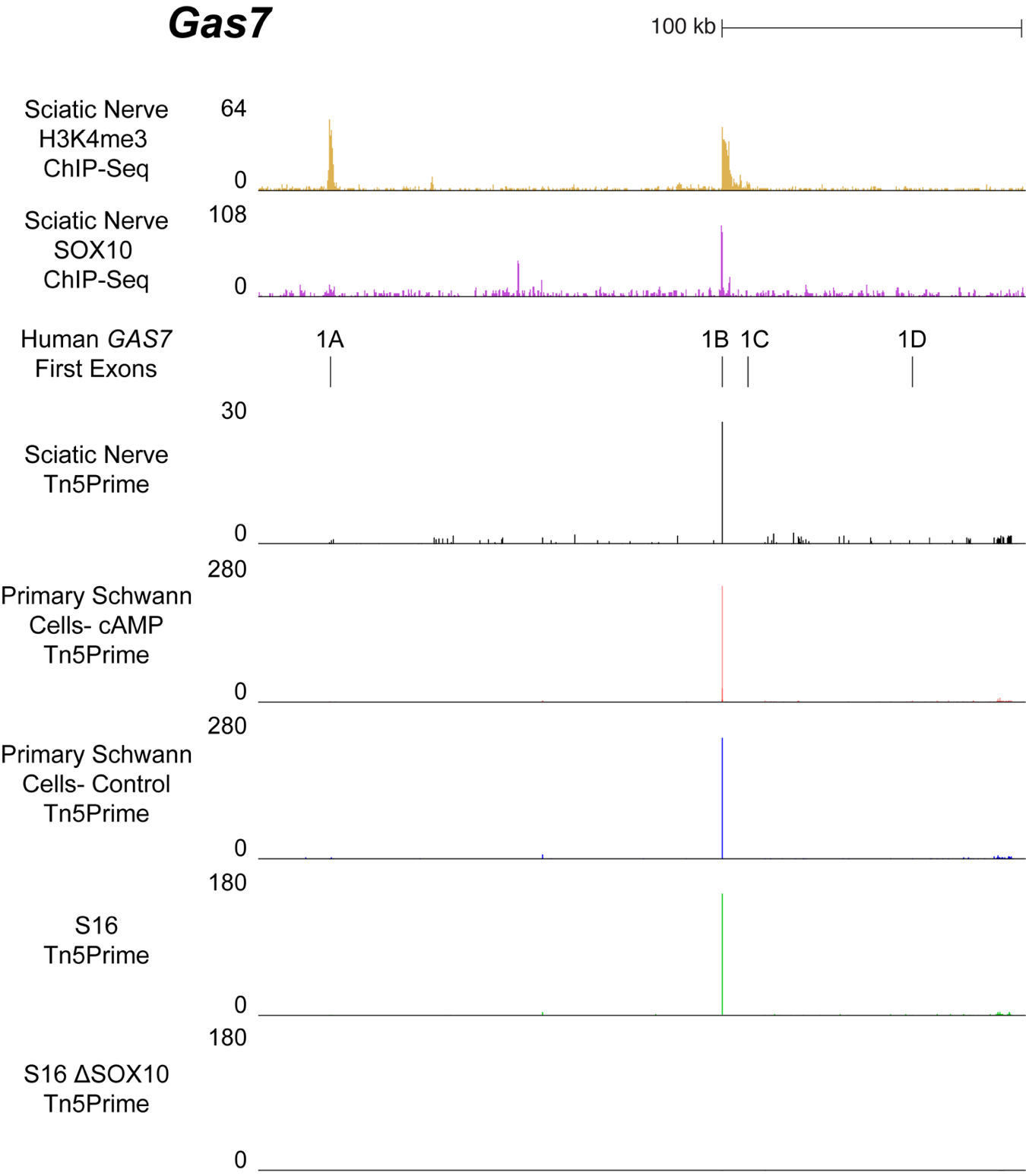
**

*Gas7* exon 1B is the predominant transcription start site utilized in Schwann cells. The rat *Gas7* genomic locus. Y-axes for H3K4me3 and SOX10 ChIP-Seq data: fold enrichment of sequencing reads above chromatin input. Below, the locations of each first exon from the human *GAS7* locus (see Supplementary Figure 8A), converted to rat genomic coordinates. Y-axes for Tn5Prime data from rat sciatic nerve, CPT-cAMP- (cAMP) and vehicle-treated (Control) primary Schwann cells, and unmodified and ΔSOX10 S16 cells: number of transcript 5’ends mapped per base, in reads per million.

**Supplementary Table 4.** Genomic elements tested for SOX10-dependent regulatory activity in luciferase assays.

| **Element** | **Coordinates (hg38)** | **Size (bp)** |
| --- | --- | --- |
| *ARPC1A* Prom 2 | chr7:99,357,845-99,358,748 | 904 |
| *CHN2* Prom 4 | chr7:29,479,363-29,480,206 | 844 |
| *DDR1* Prom 5 | chr6:30,885,974-30,886,685 | 712 |
| *GAS7* Prom 2 | chr17:10,036,393-10,037,227 | 835 |
