# Supplementary material for: SOX10-regulated promoter use defines isoform-specific gene expression in Schwann cells
Source: BMC Genomics. 2020 Aug 8;21:549. doi: 10.1186/s12864-020-06963-7 (PMC7430845; doi:10.1186/s12864-020-06963-7)
Supplement: Supplementary file 5 — Additional file 5: Supplementary Table 4. Genomic elements tested for SOX10-dependent regulatory activity in luciferase assays. [file 12864_2020_6963_MOESM5_ESM.docx]

**Supplementary Table 4.** Genomic elements tested for SOX10-dependent regulatory activity in luciferase assays.

| **Element** | **Coordinates (hg38)** | **Size (bp)** |
| --- | --- | --- |
| *ARPC1A* Prom 2 | chr7:99,357,845-99,358,748 | 904 |
| *CHN2* Prom 4 | chr7:29,479,363-29,480,206 | 844 |
| *DDR1* Prom 5 | chr6:30,885,974-30,886,685 | 712 |
| *GAS7* Prom 2 | chr17:10,036,393-10,037,227 | 835 |
